# Benchmarking imputation accuracy in the presence or absence of a reference panel

**DOI:** 10.1093/molbev/msag094

**Published:** 2026-04-10

**Authors:** Alexandros Topaloudis, Tristan Cumer, Eléonore Lavanchy, Anna Hewett, Marianne Bachmann Salvy, Anne-Lyse Ducrest, Céline Simon, Bettina Almasi, Alexandre Roulin, Olivier Delaneau, Jérôme Goudet

**Affiliations:** Department of Ecology and Evolution, University of Lausanne, Lausanne CH 1015, Switzerland; Swiss Institute of Bioinformatics, Lausanne 1011, Switzerland; Department of Ecology and Evolution, University of Lausanne, Lausanne CH 1015, Switzerland; Swiss Institute of Bioinformatics, Lausanne 1011, Switzerland; Department of Ecology and Evolution, University of Lausanne, Lausanne CH 1015, Switzerland; Swiss Institute of Bioinformatics, Lausanne 1011, Switzerland; Department of Ecology and Evolution, University of Lausanne, Lausanne CH 1015, Switzerland; Swiss Institute of Bioinformatics, Lausanne 1011, Switzerland; Department of Ecology and Evolution, University of Lausanne, Lausanne CH 1015, Switzerland; Swiss Institute of Bioinformatics, Lausanne 1011, Switzerland; Department of Ecology and Evolution, University of Lausanne, Lausanne CH 1015, Switzerland; Department of Ecology and Evolution, University of Lausanne, Lausanne CH 1015, Switzerland; Swiss Ornithological Institute, Seerose 1, Sempach CH 6204, Switzerland; Department of Ecology and Evolution, University of Lausanne, Lausanne CH 1015, Switzerland; Regeneron Genetics Center, Tarrytown, NY, USA; Department of Ecology and Evolution, University of Lausanne, Lausanne CH 1015, Switzerland; Swiss Institute of Bioinformatics, Lausanne 1011, Switzerland

**Keywords:** low-coverage, non-model species, barn owl, GWAS

## Abstract

Whole genome sequencing (WGS) of a large number of samples is costly. Solutions include targeting a proportion of the genome (eg SNP arrays) or lowering the sequencing depth (low-coverage WGS, lcWGS) but both approaches suffer from either genotype missingness or uncertainty. Genomic imputation addresses this problem by inferring missing or uncertain genotypes using a collection of high quality genomic data (reference panel). However, certain methods can impute a lcWGS dataset without a reference panel. Because generating a reference panel can be prohibitively expensive in nonmodel species, a benchmarking of the accuracy of these alternative methods of imputation can help inform study design. Here, we imputed a dataset of 2,800 barn owls (Tyto alba) sequenced in lcWGS in the presence and absence of a reference panel of 502 samples. We used 32 individuals sequenced at both high and low coverage to estimate the accuracy of each method and explored the limitations of lcWGS sample size and reference panel size. Although the best results were achieved with a large reference panel, using only a lcWGS dataset showed very accurate imputation, when over 500 samples were used and we account for missing data and low frequency alleles. In addition, imputation with or without a reference panel returned similar results in a GWAS of a polygenic trait but caution was required when comparing identified homozygous-by-descent segments. Thus, while using a reference panel remains the ideal approach, imputation in suitably large lcWGS datasets can provide sufficient accuracy given proper quality control.

## Introduction

In the last couple of decades, the development of fast and affordable DNA sequencing techniques enabled the rapid generation of genomic data which has become the backbone of research fields such as medicine and evolutionary biology. Although the cost of DNA sequencing has reduced by multiple orders of magnitude, errors that stem from sequencing technologies and downstream analyses (eg mapping, variant calling) usually necessitate sequencing a target sample in high coverage/sequencing depth (often at least 20×, Nielsen et al. 2011; Sims et al. 2014). As a consequence, sequencing can still come at a daunting cost when the number of samples required is large. Solutions to reduce these costs include sequencing a fraction of the genome (eg SNP arrays, restriction site–associated DNA sequencing [RADseq]) or sequencing the whole genome at a fraction of the depth (eg 1–2×) an approach termed low-coverage whole-genome sequencing (lcWGS) (Fuentes-Pardo and Ruzzante 2017; Benjelloun et al. 2019; Lou et al. 2021). SNP arrays have been widely used for model species but they bear a large up-front cost to develop, require a reference genome and a catalog of existing variation, and are not readily transferable across populations (Nielsen 2004; Ha et al. 2014). RADseq offers an alternative for non-model species but comes with pitfalls such as a biased sampling along the genome (Davey and Blaxter 2010) and a lower repeatability (Cumer et al. 2021), which affect downstream analyses (Lavanchy and Goudet 2023). Similarly, lcWGS has its own limitations: it requires an assembled reference genome (although reference genomes for different species are becoming increasingly available; Rhie et al. 2021; Gupta 2022; The Darwin Tree of Life Project Consortium 2022) and comes with significant genotypic uncertainty and high levels of missing data. In contrast, lcWGS enables the de-novo discovery of variation in a sample of individuals (Le and Durbin 2011; Martin et al. 2021) and specialized methods and tools exist that can account for the substantial genotypic uncertainty (eg ANGSD, Korneliussen et al. 2014) making lcWGS an attractive solution for non-model species (Lou et al. 2021).

To overcome the shortcomings of both SNP arrays and lcWGS, sequencing data can be coupled with genomic imputation, which generates genome-wide data (often phased) from incomplete observations, providing access to a powerful set of analyses (Marchini and Howie 2010; Das et al. 2018). Usually, genomic imputation infers unobserved genotypes, such as variants uncertain or missing in lcWGS by using a reference set of high-quality haplotypes (a reference panel). The principles behind imputation lie in population genetic theory (Li and Stephens 2003; Browning 2008; Marchini and Howie 2010; Das et al. 2018) and utilize the fact that even distantly related individuals in a population share segments along their genome that are identical-by-descent. Therefore, the unobserved haplotypes of a focal sample can be “pieced together” by using the scarce genotyped data and identifying the corresponding observed haplotypes present in the reference panel. The algorithms account for differences due to mutation and allow haplotype switching due to recombination events. Different versions of imputation have been implemented using different input data and assumptions (eg BEAGLE, Browning et al. 2018; GLIMPSE, Rubinacci et al. 2020; QUILT, Davies et al. 2021; IMPUTE5, Rubinacci et al. 2021). Because they rely on linkage disequilibrium (LD), these methods can be collectively classified under the term “linkage-disequilibrium imputation methods”. In addition to the methods mentioned above, some implementations can take advantage of specialized resources available in the target species. For example in livestock species with large documented pedigrees specialized imputation software can use relatedness along a pedigree to impute target individuals (eg (Hickey et al. 2012; Cheung et al. 2013; Sargolzaei et al. 2014).

Estimated imputation accuracy is often very high for inferences in humans, livestock, ancient DNA (aDNA) datasets as well as in simulated data (Pasaniuc et al. 2012; Lloret-Villas et al. 2023; Sousa da Mota et al. 2023; Erven et al. 2024). Specifically, imputation performance is excellent for alleles at intermediate frequencies but diminishes for rare alleles, although including pedigree information can improve imputation of low-frequency alleles (Liu et al. 2019). The negative correlation of imputation accuracy and allele frequency stems from rare alleles being found in only few copies in the reference panel haplotypes and not being sufficiently tagged by surrounding variation making them harder to infer. In addition to allele frequencies, imputation performance is sensitive to the composition of the reference panel used. It has been shown that imputation performs best when the reference panel used is large, well-phased and is representative of the haplotypic diversity in the target population (Das et al. 2018; De Marino et al. 2022; Garcia et al. 2022). These findings have spurred the development of large reference panels in humans and other model species, where whole-genome sequences of hundreds of thousands of samples are routinely used to impute a scarcer set of typed variation (McCarthy et al. 2016; Rowan et al. 2019; Wang et al. 2022; Shi et al. 2024).

When a reference panel is not available, imputation can be performed using only low-coverage whole-genome sequencing data. Such approaches are similar to LD-based imputation methods but make assumptions about the underlying haplotypes that have to be pieced together by the lcWGS dataset instead of being observed in a reference panel. Existing implementations include BEAGLE (Browning and Browning 2007) and STITCH (Davies et al. 2016). The resulting accuracy of reference-free imputation is promising and can pave the way for the wider adoption of lcWGS in wild populations without the need for a costly reference panel (Delaneau and Marchini 2014; Davies et al. 2016; Teng et al. 2022; Yang et al. 2024). Nevertheless, few empirical studies on non-model species have used imputation of lcWGS in wild populations with or without reference panels (Fuller et al. 2020; Gao et al. 2021; Enbody et al. 2023; Hooper et al. 2024; Justen et al. 2024; Landis et al. 2024). This stems from a lack of available genomic resources but also from a shortage of thorough validation of imputation methods in wild populations, which might generate uncertainty about the accuracy of the resulting genotypes. To date, there are very few tests of the accuracy of imputation in wild populations (Watowich et al. 2023; Tan et al. 2025), with none specifically comparing the presence and absence of a reference panel. Although comparisons exist for humans and livestock (Shi et al. 2019; Teng et al. 2022; Wang et al. 2024), the low effective population size, high LD, and unique mating schemes practiced in selective breeding can inflate imputation accuracy and these results are not readily transferable to wild populations. Furthermore, most benchmarking studies rely on simulated data as the ground truth or on artificially downsampling high coverage samples to generate a lcWGS dataset in-silico, which might not accurately reflect the specificities of DNA sequencing.

**Figure 1:**
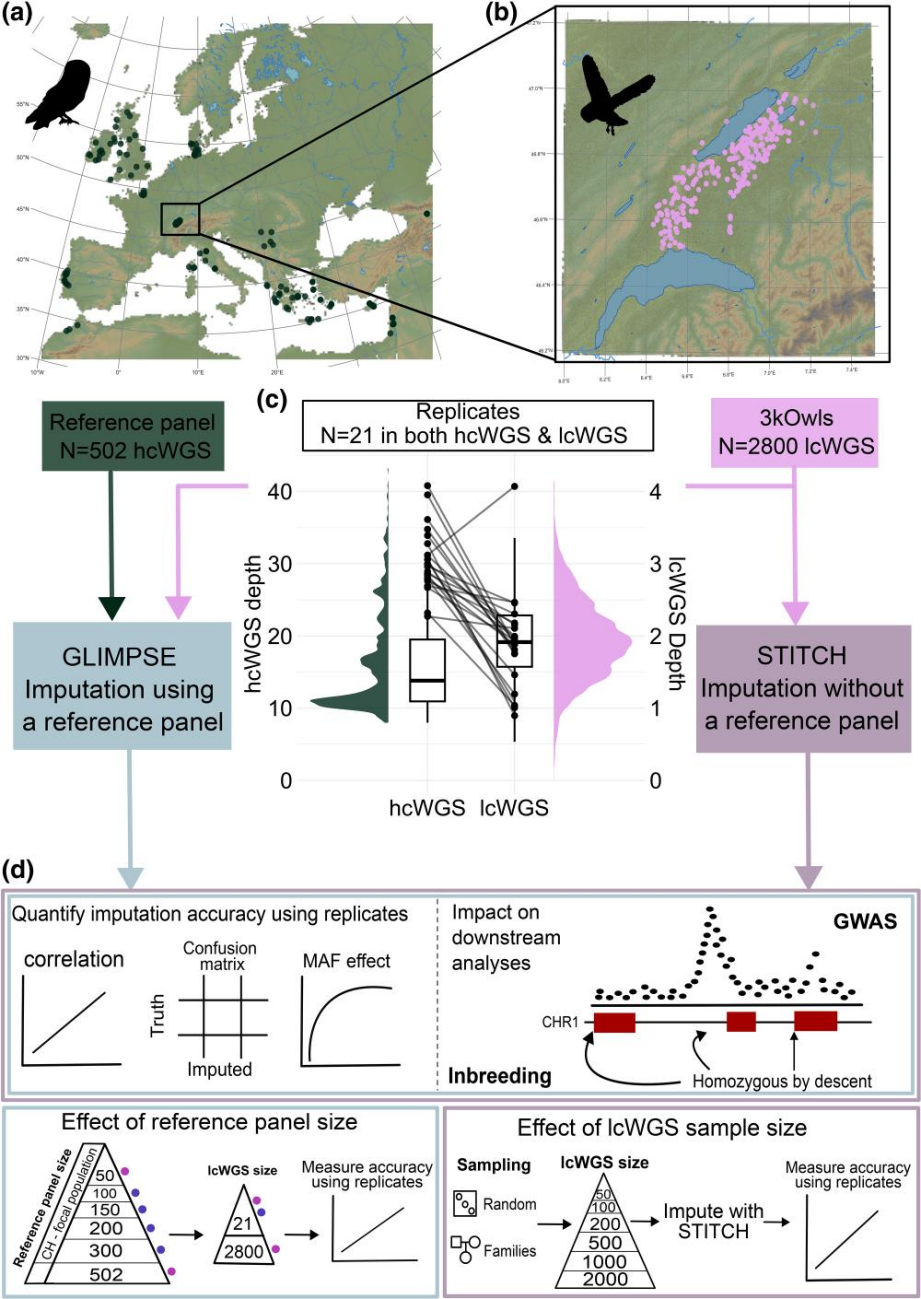
A graphical abstract of the study. (a) The barn owl (*Tyto alba*) reference panel of 502 samples sequenced in medium to high-coverage WGS (hcWGS), 346 of which come from the focal long-term study in Western Switzerland (CH). (b) 2,800 samples from the long-term study in Western Switzerland sequenced at low-coverage WGS (lcWGS). (c) Sequencing depth of the datasets used. Left: sequencing depth in the reference panel; right: sequencing depth of the lcWGS. Black dots represent the 21 high-depth replicate samples sequenced in both datasets. (d) Top left: the accuracy of imputation with GLIMPSE and STITCH quantified using the replicate samples including the effect of minor allele frequency (MAF). Top right: sketch of downstream analyses like a polygenic trait GWAS and inbreeding along the genome. Bottom left: The effect of reducing the reference panel size. Colored dots show which reference panel size was used to impute which lcWGS size. For a reference panel of 50 samples, we tested lcWGS sizes of 21; 100; 200; 500; 1,000; 2,000, and 2,800. Bottom right: The effect of lcWGS sample size and sampling strategy. Owl silhouettes from PhyloPic.

To address this, we quantify the accuracy of imputation in a dataset of lcWGS in a wild population of barn owls (*Tyto alba*) where a reference panel is also available (Fig. 1). We use 2,800 samples recently sequenced in lcWGS (average depth = 2×—Fig. 1b) (Cumer et al. 2025) and impute them with or without the use of a reference panel of 502 medium to high-coverage (hcWGS) individual whole genomes (Fig. 1a) using GLIMPSE and STITCH, respectively. We measure the imputation accuracy of each approach using 21 individuals sequenced both at high and low coverage (Fig. 1c). Further, we evaluate the effect of reference panel size, and lcWGS sample size on imputation accuracy and evaluate the downstream impact on estimating inbreeding along the genome and carrying out a GWAS for a polygenic trait (Fig. 1d). We explore the benefits and limitations of the two approaches and provide guidelines on using lcWGS coupled with imputation.

## Results

### Comparison of approaches

We imputed 2,800 low-coverage whole-genome sequences (lcWGS) with and without the use of 502 medium to high coverage WGS (hcWGS, reference panel) (Fig. 1). When making use of the hcWGS, GLIMPSE (reference-based imputation hereafter) imputed a set of 10,102,233 bi-allelic SNPs identified in the reference panel and genotyped in the lcWGS data. When using only the lcWGS dataset, we used STITCH (reference-free imputation hereafter) which imputed a set of 21,732,187 bi-allelic SNPs de-novo identified in the 2,800 lcWGS samples.

This work is a comparison of two approaches, imputation with a reference panel and imputation without a reference panel. Any difference in performance stems from both algorithmic differences between GLIMPSE and STITCH and the presence/absence of a reference panel. We also tested imputation using QUILT, an extension of the STITCH algorithm that uses a reference panel. Imputation accuracy using QUILT was comparable to GLIMPSE, the latter performing slightly better probably due to the sequencing depth of the lcWGS dataset (Figure S1). Since both QUILT and GLIMPSE performed similarly, the central contrast in this work is the comparison of imputation with versus without a reference panel, rather than one between the specific algorithms implemented.

For validation we used a set of 32 samples sequenced both at high and low coverage, which we call “replicates.” All high coverage genotypes of the replicates were removed from the reference panel during imputation with GLIMPSE (see Methods). One was an error in labeling and was discarded. Ten of these were sequenced at lower sequencing depth in the high coverage “truth set” (mean depth 11×; range 8.4–17.3×; hereafter “intermediate-depth replicates”), while the rest (*n* = 21) were sequenced in higher sequencing depth (mean depth = 30.5, range = 22.7 to 40.8, hereafter “high-depth replicates,” Fig. 1c). The intermediate-depth replicates showed a pattern of very high sensitivity and low precision (see methods) only in the heterozygote genotypes (Figures S2 and S3). This pattern could stem from missing heterozygous calls in the “truth dataset” which were then correctly inferred when imputation was used but classified as errors in the comparison. Consistent with this hypothesis, the ten intermediate-depth replicates exhibited less heterozygous sites than the 21 higher-depth ones (Figure S4). For this reason, these ten individuals were subsequently excluded from the replicate set used to validate the imputation results. Thus, unless stated otherwise, the 21 high-depth replicates are considered the ground truth against which all comparisons are made.

We tested the effect of filtering on the accuracy of imputation by using only SNPs with an information score (the estimated imputation accuracy from the software; INFO) larger than 0.6 or 0.8 compared to using the unfiltered set of variants. Both cutoffs increased the quality of imputation at the cost of reducing the number of variants remaining (Figure S5). Filtering at INFO = 0.8 reduced the number of variants retained in GLIMPSE and STITCH to 94% and 95% of the method's total, respectively (Table S1). For the rest of the results, we use an imputation accuracy filter of 0.8 leaving 7,865,446 SNPs at the overlap of GLIMPSE and STITCH. Results for different filtering cutoffs can be found in Figures S6 and S7.

### Overall imputation accuracy

**Figure 2:**
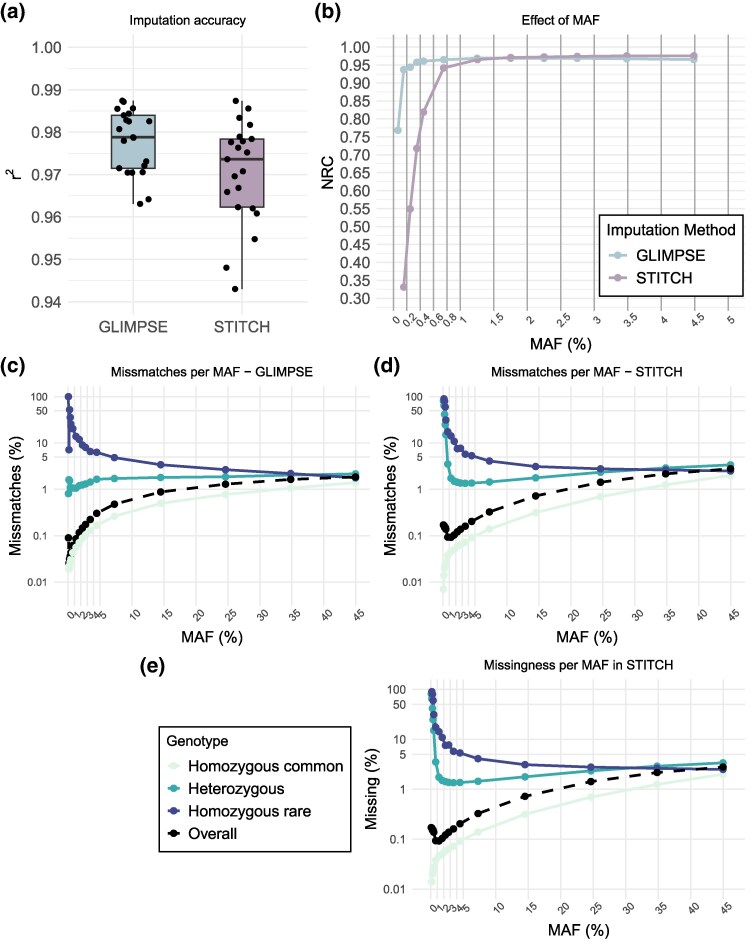
The accuracy of imputation with and without a reference panel. (a) the squared correlation (imputation accuracy) per sample between the “truth” (high-coverage data) and the low-coverage WGS dataset imputed by using a reference panel with GLIMPSE or by using only the lcWGS dataset with STITCH. Each point is one of the 21 replicate individuals. (b) Non-reference concordance (NRC) per minor allele frequency (MAF) bin. Each point represents the mean in the specific allele frequency bin. Allele frequency for the rare allele was defined in the GLIMPSE dataset of 2,800 samples. (c and d) The misclassification profile of each genotype class across each allele frequency bin for GLIMPSE (c) and STITCH D). (e) Missing data profile for STITCH for each genotype class across each allele frequency bin. Legend shared across panels c, d, and e.

The per sample accuracy of imputation (quantified as the squared Pearson correlation, *r*^2^, between the high coverage and imputed genotypes of each replicate) was very high (>0.95) regardless of whether a reference panel was used or not (Fig. 2a). However, reference-based imputation showed significantly higher imputation accuracy (mean = 0.978; SD = 0.008) with lower variation among individuals, compared to reference-free imputation (mean = 0.970; SD = 0.012; Wilcoxon rank sum exact test; *W* = 305, *P* = 0.034).

To explain the among-sample variation in imputation accuracy we regressed the reference-based imputation accuracy of the high-depth replicates on the sequencing depth in the truth and the low-coverage set along with the average relatedness in the reference panel and the lcWGS dataset (Figures S8 and S9). The model explained 8.5% (adjusted *R*^2^ = 0.085) of the variation in imputation accuracy and no factor was significant. For reference-free imputation we repeated the same model excluding the relatedness to the reference panel. This model explained approximately 27% of the per-sample variation in imputation accuracy (adjusted *R*^2^ = 0.269). The only statistically significant factor in the model was the low-coverage sequencing depth of the sample (β = 0.016, *P* < 0.05). In both models when including the intermediate-depth replicates, there was a strong effect of the high-coverage sequencing depth, in line with the assumption that lower depth samples underestimate imputation accuracy due to errors in the truth set (Figures S10 and S11).

Imputing rare alleles is notoriously difficult (Marchini and Howie 2010). To measure performance across allele frequencies, we quantified the non-reference concordance (NRC), which is better suited for low-frequency alleles than imputation accuracy where the multiple homozygotes for the common allele will bias the results (Li et al. 2011; Bizon et al. 2014). When referring to a reference allele, we always refer to the most common allele at each locus. Both approaches showed very high NRC in allele frequencies above 1% to 2% (Fig. 2b). When using only lcWGS data with STITCH, NRC dropped slightly for variants with a minor allele frequency below 1% and plummeted below 0.8%. The use of a reference panel with GLIMPSE showed accurate estimation across most allele frequency classes but exhibited a sharp decrease in allele frequencies below 0.2%.

To identify the origin of imputation errors, we measured the proportion of each genotype misclassified with each method along the allele frequency bins (Fig. 2c and 2d). Both methods had a similar misclassification profile for all genotype classes, with STITCH exhibiting an increased overall rate across all frequency bins and genotype classes along with a higher misclassification of heterozygote genotypes in rare alleles (<1%). As expected, heterozygotes were misclassified as either homozygote genotypes and homozygotes for the alternative allele are almost always misclassified as heterozygotes (Figure S12).

When using STITCH some genotypes are set to missing when the inferred genotype probability lies below a certain threshold (0.9 by default). This behavior leads to a proportion of missing data per sample (average across 2,800 individuals: 1.5%, range: 0.7% to 26.8%) which correlates strongly and negatively with the lcWGS depth at which each sample was sequenced (Figure S13). In addition, overall missingness increased as the allele frequency increased (Fig. 2e). For heterozygotes, high missingness was observed mostly in rare alleles and it plateaued to about 5% in allele frequencies larger than 2%. The genotype class with the largest proportion of missing data is the homozygotes for the rare allele. However, because STITCH reports the genotype probability, even when the called genotype is missing, we could compare the full genotype probabilities (no missing data) with the true genotype of the replicates. The accuracy of imputation with STITCH per sample when using the genotype probabilities (including the non-called genotypes) dropped on average by 0.13% (using genotype calls, mean = 97%; using genotype probabilities, mean = 96.9%) (Figure S14).

### The effect of reference panel size

**Figure 3:**
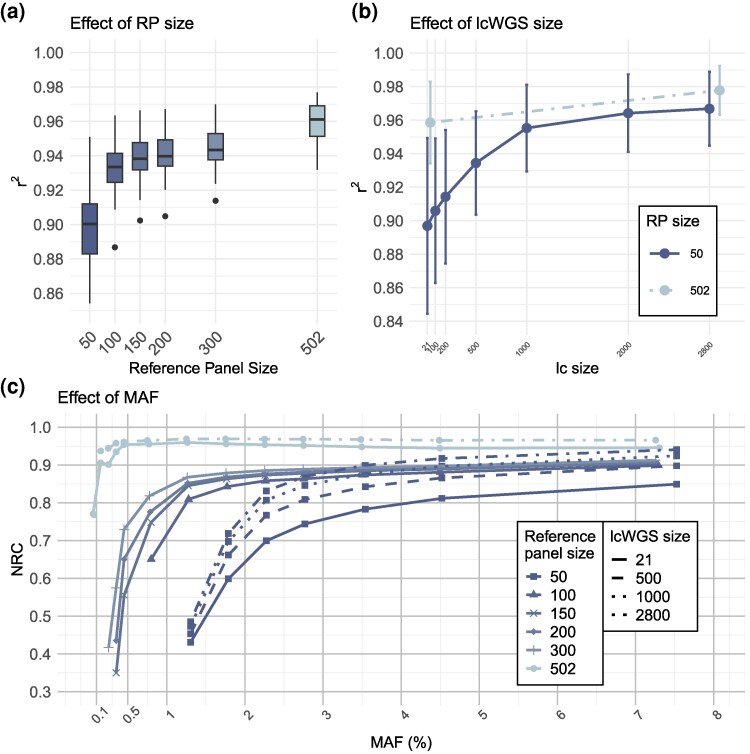
The effect of sample size on imputation accuracy when using a reference panel. (a) The per sample imputation accuracy using different reference panel sizes when imputing the 21 replicate lcWGS samples. (b) The effect of increasing the lcWGS dataset. Dash-dot line connects the imputation accuracy of 21 and 2,800 lcWGS samples when using the full reference panel. (c) The non-reference concordance (NRC) across minor allele frequency (MAF) bins for different reference panel sizes. Each RP dataset (except for the full one of 502 samples) is truncated at a frequency of 1 over twice its sample size. RP: reference panel. lcWGS: low-coverage whole-genome sequencing dataset.

To address the effect of reference panel size, we ran GLIMPSE using a set of 300, 200, 150, 100, and 50 samples from the focal population of Western Switzerland. We chose these samples by prioritizing founders and individuals with many descendants and re-phased them to generate realistic datasets (Figure S15). Using the above reference panel sample sizes, we imputed only the 21 high-depth replicates. We also imputed these 21 samples, without other lcWGS samples, using the full reference panel of 502 individuals. We observed increasing imputation accuracy with increasing reference panel size (Fig. 3a). All reference panel sizes showed an average imputation accuracy above 0.9, with a reference pane size above 200 samples returning more than 0.95 imputation accuracy per sample. The largest benefit was seen when increasing the reference panel size from 50 to 200 individuals (1.15% accuracy increase per 50 samples added) while further increase provided reduced benefits (0.23% accuracy increase per 50 samples).

We tested the effect of increasing the lcWGS sample size when imputing with a given reference panel size and found a considerable positive effect on overall imputation accuracy (Fig. 3b). Using the full reference panel, we imputed 21 and 2,800 lcWGS and using a reference of 50 samples, we imputed sizes of 100; 200; 500; 1,000; 2,000, and 2,800 lcWGS. While the full reference panel using 2,800 lcWGS samples, instead of only the 21, led to a 1.5% increase, the effect was larger in the smaller reference panel size. In that case the same increase in lcWGS sample size returned a 6.5% absolute increase in imputation accuracy. The gain of increasing the lcWGS dataset diminished after approximate 1,000 samples were added.

Non-reference concordance increased with increasing reference panel size across all allele frequencies (Fig. 3c). The high NRC at very rare alleles was a feature exclusive to the full reference panel, as all other sizes showed a decrease below a MAF of 1%. In addition, adding more lcWGS samples increased NRC but was still limited at lower allele frequencies (non-full lines in Fig. 3b).

### The effect of lcWGS sample size

**Figure 4:**
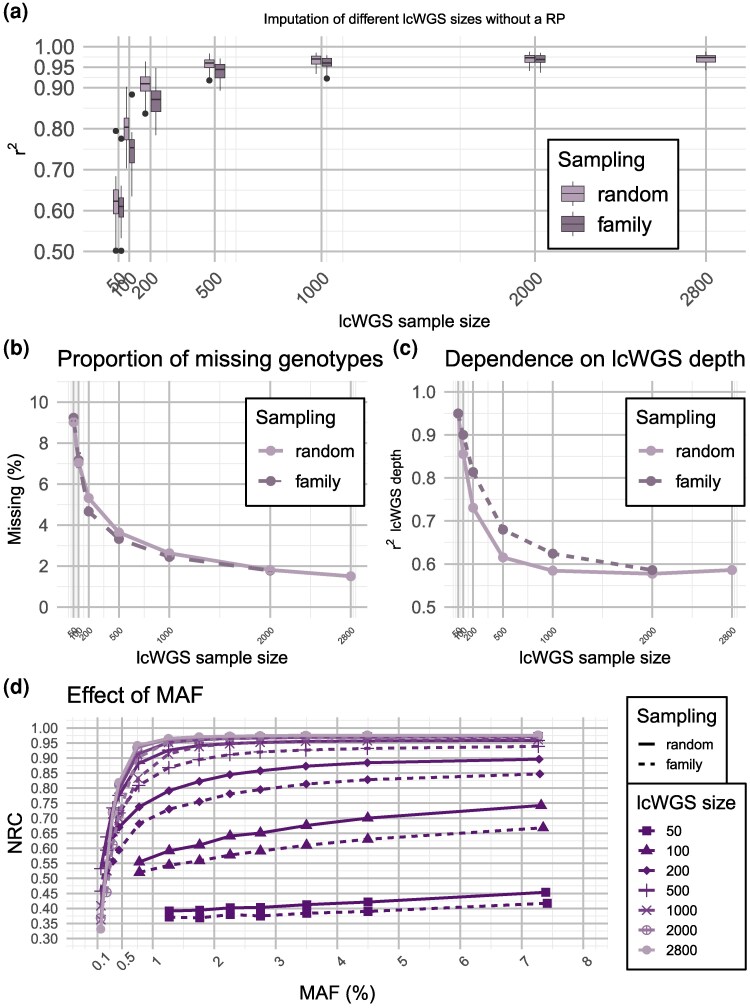
The effect of low-coverage sample size on imputation accuracy. (a) The imputation accuracy per sample in the 21 replicates using a different number of low-coverage whole-genome sequences (lcWGS) in STITCH. (b) The average genotype missingness (95% CI shown as error bars) in genotypes imputed by STITCH across all samples in each subset. (c) The correlation of imputation accuracy and lcWGS depth for the 21 replicates for different sizes of the lcWGS dataset, a larger correlation shows larger dependence on the underlying sequencing depth of each sample. (d) The non-reference concordance across minor allele frequency bins for different lcWGS sizes. Each lcWGS dataset (except for the full one of 2,800 samples) is truncated at a frequency of 1 over twice its sample size. For all panels the subsampled dataset was generated by either random sampling or by sampling whole families of individuals.

We tested imputation accuracy in subsets of the lcWGS without using a reference panel, always including the high-depth replicates, down to a size of 50, 100, 200, 500, 1,000, and 2,000 individuals. We chose these individuals either by sampling randomly across our dataset or by sampling full families, representing two realistic sampling schemes that might be encountered in a study system and identified variants de-novo (number of variants per subset can be found in Figure S16). We then imputed these datasets using STITCH. The accuracy of imputation benefited from increasing sample size (Fig. 4a) with diminishing returns in accuracy gain when using more than 500 samples, which achieved an imputation accuracy of approximately 0.95 on average.

Since we observed missing data and a dependence of per-sample imputation accuracy on lcWGS sequencing depth, we quantified these aspects in the smaller lcWGS sample sizes. The percentage of missing data decreased with increasing sample size, even after reaching 1,000 samples (Fig. 4b). To measure the dependence on lcWGS depth, we estimated the correlation of imputation accuracy and lcWGS depth. A higher correlation implies a larger effect of lcWGS depth on performance. The correlation for small sample sizes (*n* = 50, 100) was almost complete (>0.9) implying that imputation was rather ineffective at these sample sizes, and most genotypes resulted from observed data (Fig. 4c). On the other hand, above 500 to 1,000 samples, this metric achieved its smallest observed value (approximately 0.6). Finally, the non-reference concordance across allele frequency bins increased with increasing sample size and was very similar from 500 samples and practically indistinguishable from 1,000 samples at 2× depth (Fig. 4d). In all comparisons, a random subset of individuals performed better than a family stratified one which can be explained from the stochastic inclusion of more relatives of the replicate individuals in the random dataset (Figure S17).

### Impact on downstream analyses

**Figure 5:**
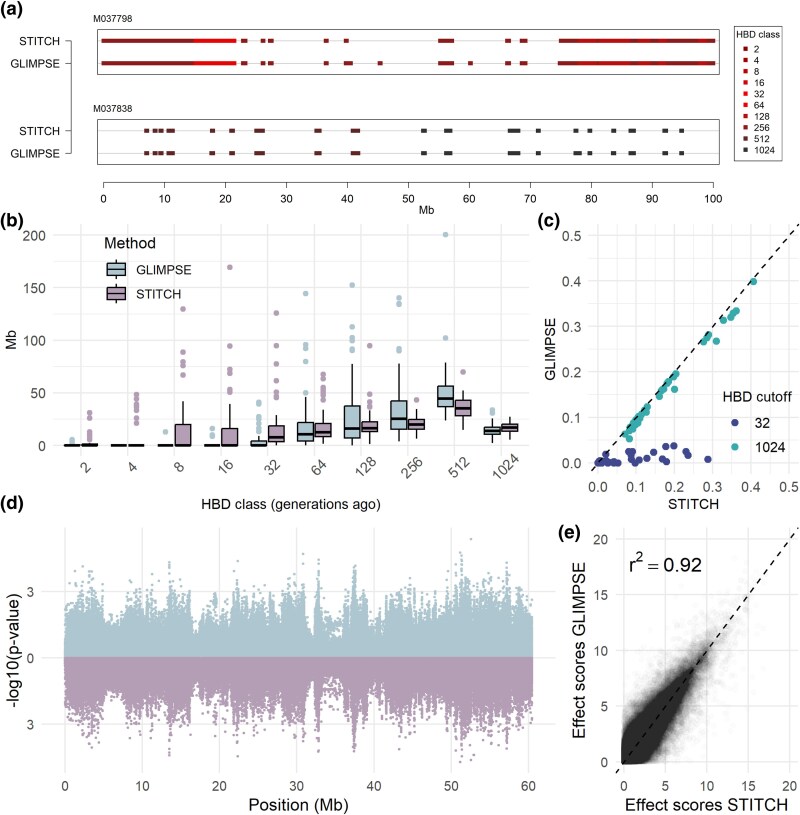
Downstream analyses in the imputed datasets. (a) The homozygous-by-descent (HBD) segments inferred for an inbred (M037798) and a non-inbred sample (M037838) along the first 100 Mb of the genome. HBD classes correspond to the two homozygous segments sharing a common ancestor that many generations ago. (b) The distribution of the length of all HBD segments assigned to a specific class using the 51 samples when imputed with reference panel (GLIMPSE) and when imputed only with lcWGS (STITCH). (c) The proportion of the genome annotated in being HBD up to a certain number of generations ago between the two imputed datasets for the 51 individuals. (d) Manhattan plot of −log10 *P* values from a GWAS on adult tarsus length with either imputed dataset (reference-based imputation above, reference-free imputation below) for an example linkage group (LG2) (e) Plot of effect scores among the two methods for the same SNPs as the *P*-values in panel D.

The homozygosity along the genome of an individual is a useful metric that can provide important insights about the inbreeding state of wild populations with implications for conservation. To quantify the performance of each dataset, we annotated homozygous-by-descent (HBD, equivalent to ROH) regions along the genome using the R package RzooRoH (Bertrand et al. 2019). The overall distribution of the ROH segments was very similar between datasets, with reference-based imputation identifying slightly more short ROH segments where reference-free does not (example region in Fig. 5a). One major difference was the assignment of ROH segments into different length classes corresponding to a different age of the common ancestor. Specifically, using the genotypes imputed using only the lcWGS dataset, RzooRoH assigned more segments into younger classes (age < 32 generations ago) compared to when using a reference panel (Fig. 5b). As a consequence, when calculating genome-wide inbreeding coefficients using only young ROH segments (<32 generations ago) the results differed between the two methods (Fig. 5c). In contrast, including older ROH classes resulted in equivalent inbreeding coefficients between the two methods (Fig. 5c).

Imputed data are often used in a genome-wide association study (GWAS) to identify the regions of the genome affecting a phenotype (the trait's architecture). To test the performance of the different imputation methods on a GWAS, we looked for an association of the imputed genotypes with the length of the left tarsus, a trait with an intermediate heritability in adult birds (*h*^2^ = 0.216, Hewett et al. 2025). We selected only individuals with the phenotype measured at the adult stage of their life (*n* = 1,980) and fitted their sex as a fixed effect. For both imputed datasets we fitted a linear mixed model including the allele sharing genomic relationship matrix as a random effect to account for confounding population and family structure. The results were very similar between reference-based and reference-free imputation and both the inferred effect scores and *P*-values were highly correlated (Squared Pearson's correlation for effect scores = 0.92; *P*-values = 0.87; an example linkage group can be seen Fig. 5d and 5e). Full results in Figure S18.

## Discussion

In this study, we compare imputation methods with and without a reference panel to establish best practices for imputation in wild populations. Using samples sequenced in both low and high coverage as a validation set, we demonstrate that imputation of 2,800 lcWGS samples is highly accurate when a reference panel of 502 individuals is used. Reducing the reference panel size to 50 individuals from the focal population still achieves accuracy larger than 90% and increasing either the reference panel size or the lcWGS dataset improves performance. In the absence of a reference panel, imputation of the 2,800 lcWGS produces comparable accuracy to that achieved with a reference panel of 500 samples, though it struggles with low-frequency alleles (<1%). For smaller lcWGS data sets, imputation accuracy can be high from 500 samples, provided rigorous quality control is applied. When using the entire lcWGS dataset, a polygenic GWAS revealed minimal differences between imputations with or without a reference panel, but some caution was required when looking into runs of homozygosity (ROH). We conclude with recommendations for using imputation in lcWGS studies of wild populations.

### Imputation with a reference panel

Imputation using a reference panel has been shown to be very accurate in humans, domestic animals, crop plants, and simulated data (Swarts et al. 2014; Das et al. 2018; Deng et al. 2022; Teng et al. 2022). Recently, it has been demonstrated that it performs well in wild populations (Watowich et al. 2023; Tan et al. 2025). Watowich et al. (2023) quantified imputation accuracy in two empirical datasets of different species, one with a large reference panel (*n* = 741; rhesus macaques *Macaca mulatta*) and one with a small reference panel (*n* = 68; gelada monkeys *Theropithecus gelada*). The authors found accurate imputation in both datasets, even for the small reference panel sizes in the gelada dataset (*r*^2^ = 0.87 and 0.96 for allele frequencies in the range of 1–5% and 10–50%, respectively). In addition, Tan et al. (2025) attained very high imputation accuracy by using a limited number of individuals as a reference panel (*n* = 29) (>0.9) in the hihi/stitchbird (*Notiomystis cincta*). However, the gelada dataset harbors a small number of polymorphic sites, low heterozygosity and elevated ROH (Chiou et al. 2022; Watowich et al. 2023) and the hihi has notoriously low diversity due to a dramatic past bottleneck event leaving approximately half of the genome homozygous by descent (de Villemereuil et al. 2019; Duntsch et al. 2023). The barn owl dataset used here displays a larger diversity (average nucleotide diversity 0.0015) due to a well-connected continental population and rare occurrence of inbreeding (Cumer et al. 2022a; Hewett et al. 2025). It also exhibits linkage disequilibrium (LD) patterns characteristic of birds, with microchromosomes of elevated recombination rates (low LD) and large chromosomes of diverse recombination landscapes (Topaloudis et al. 2025). Therefore, it represents a distinct parameter space of the diversity and LD values studied so far. Based on our investigation we show that imputation using a large reference panel (502 individuals) coupled with 2X lcWGS can generate imputation with accuracy greater than 0.97 (*r*^2^ > 0.97) even for a species with genetic diversity larger than those studied previously and this result can extend to low-frequency alleles (MAF > 0.2%).

An important consideration for non-model species is the size of the reference panel. We found high imputation accuracy (*r*^2^ > 0.9) even when reducing the size of the reference panel to 50 individuals, and while any increase beyond 50 samples increased the resulting accuracy, diminishing returns were observed above 200 individuals. On the other hand, reducing the size of the reference panel to 300 or below, reduced performance in rare alleles (MAF < 1%). Therefore, while imputation accuracy can be high, the performance in rare alleles will depend on large sample sizes and accurate phasing. Still, we showed that despite substantial genetic diversity in the barn owl, we also find accurate imputation with a reference panel of 50 individuals (Watowich et al. 2023; Tan et al. 2025), a promising result for studies in non-model species.

Concerning the composition of the reference panel, despite a large number of individuals (*n* = 346) of the focal Swiss population, there was improved imputation accuracy when including samples from other populations (ie using the full reference panel). This result is in line with studies in humans (Purnomo et al. 2025), dairy cattle (Lloret-Villas et al. 2023), and Nile tilapia (*Oreochromis niloticus*) (Garcia et al. 2022), where the inclusion of a large and diverse reference panel outperforms population-specific panels which lack an exhaustive representation of the haplotypes present in the target population (but see (Dekeyser et al. 2023) for an investigation into the possible caveats of this practice). Similar results are found by studies in aDNA, where relevant reference panels might be impossible to collect (Sousa da Mota et al. 2023; Bougiouri et al. 2025; Erven et al. 2024). Our results therefore support the conclusion that even in the presence of a genetically relevant reference panel, combining all available resources can improve the imputation accuracy.

A less discussed factor influencing imputation accuracy is the size of the lcWGS dataset. Because reference-based software use, the imputed lcWGS haplotypes as part of the reference panel (Rubinacci et al. 2021), increasing the lcWGS dataset can also provide an increase in imputation accuracy. We observed a 1.6% increase on average when imputing the full set of 2,800 lcWGS versus imputing only the 21 lcWGS replicates using the full reference panel. The effect was larger in the 50-samples reference panel because with small reference panel sizes, the haplotypic diversity of the population might not be sufficiently sampled. Thus, in all cases and especially when the reference panel is small, imputing samples using the full lcWGS dataset will exhibit the best performance.

Finally, an important finding was the impact of sequencing depth of the “truth set” in the resulting imputation accuracy. Through our diverse set of replicate individuals, we found that samples sequenced at intermediate depth (<20×) in the truth set, showed a lower imputation accuracy than samples sequenced at higher depth (>20×). This is due to a reduction of the heterozygous genotypes in the “truth set,” a well-known caveat of low to medium sequencing depth (Nielsen et al. 2011; Kardos and Waples 2024). Interestingly, these heterozygotes were inferred in the imputed dataset showing that imputation with a reference panel can outperform genotype calling at intermediate sequencing depth. Based on this finding, we suggest using the individuals sequenced at highest depth (>20×) in each study to validate the imputation results, and build a reference panel. Failure to do so might underestimate the accuracy of imputation.

Assessing imputation accuracy using a real dataset can incorporate realistic variation and errors in the data generating process (library preparation, sequencing depth, mapping, etc.) but explores a parameter space defined by the particularities of the study system. What characterizes our dataset is a high degree of relatedness among individuals of the reference panel, the lcWGS dataset and between the two (Figures S19 and S20). High relatedness in the reference panel can improve haplotype phasing (Glusman et al. 2014) but will decrease the number of observed haplotypes, reducing the “effective sample size,” which might explain why including non-Swiss samples marginally improved imputation accuracy. Relatedness within the lcWGS dataset can provide an increase in imputation accuracy when using a large lcWGS dataset. Such a structure might be responsible for the high imputation accuracy seen when using the full lcWGS dataset instead of only the 21 replicates. Finally, relatedness between the lcWGS and the reference panel can similarly boost imputation accuracy by long haplotype sharing among close relatives. The latter might be partially responsible for the very accurate imputation of rare alleles, a behavior expected when using pedigree-based imputation methods (Liu et al. 2019) but here observed using GLIMPSE, a linkage-disequilibrium (LD)-based method. In summary, while some aspects of our results might be influenced by the specificities of our study species, the broad findings should hold for most wild species. In addition, in long-term monitoring studies where similar familial structures might emerge, we illustrate the benefits of imputation even for low-frequency alleles, when using a reference panel with standard LD-based imputation methods.

### Imputation without a reference panel

Imputation of lcWGS in the absence of a reference panel, can be a competitive alternative when a reference panel is not available, although imputation accuracy will depend on the size and the depth of the lcWGS dataset. In past studies as well as in our own results, there appears to be a “critical size” below which genotype refinement is mostly inaccurate, and above which diminishing returns are observed. Importantly, this critical size should be dependent on the underlying genetic diversity of the organism and the lcWGS sequencing depth. In species with low genetic diversity, maximum accuracy can be achieved with smaller sizes and sequencing depths (eg 200 dairy cattle at 1× (Teng et al. 2022), 500 mice at 0.1× (Davies et al. 2016). On the contrary, in more genetically diverse species the sample size and sequencing depth required to achieve similar imputation quality is often larger (eg 500 owls at 2× (this study), at least 1,000 human samples at 1× (Davies et al. 2016), and 500 samples at 2× in a simulation study with intermediate diversity and LD (Lou et al. 2021)). Past comparisons show that the maximum attainable accuracy can be limited by lcWGS depth, meaning that a lcWGS dataset of 0.5× might never achieve the same imputation accuracy of a dataset in 2× regardless of sample size (Davies et al. 2016; Lou et al. 2021; Yang et al. 2024). In our investigation, variation in sequencing depth (a realistic expectation in any sequencing experiment) resulted in varying coverage for our replicate samples. We found that individuals with a lower coverage depth had a smaller imputation accuracy, an effect which becomes more pronounced with fewer individuals sequenced. From a practical perspective, because DNA extraction and library preparation will be the major per-sample costs in lcWGS studies, increasing sequencing depth to ∼2.0× represents a modest marginal cost while improving imputation accuracy and thus effective GWAS power. Therefore, our results prove that imputation without a reference panel can be accurate, provided that the dataset meets minimum requirements in number of samples and sequencing depth, which in our case were >500 and 2×, respectively.

Despite its overall accuracy, imputation using only lcWGS had two main shortcomings: poor performance for rare alleles and a proportion of missing data. Lower performance in rare alleles (MAF < 1%) is an expected outcome of imputation without a large reference panel and while filtering out rare alleles might introduce biases (eg in kinship estimation (Weir and Goudet 2017)) it is common practice, especially for GWAS where power is low when alleles are rare. On the contrary, the missing data are an intended behavior using default parameters in STITCH, which does not call genotypes with a genotype probability smaller than 0.9. This behavior has spurred previous studies to suggest imputing the sporadic missing data after STITCH, ie using BEAGLE (Teng et al. 2022; Vi et al. 2025). Here, we show that missing genotypes can be inferred from genotype probabilities with minimal accuracy loss eliminating the necessity for a second imputation step. Notably, both missingness and poor imputation in low-frequency alleles are exaggerated in small lcWGS datasets so studies planning to use a small lcWGS dataset without a reference panel should address these issues. We assume a valid solution to be a relevant MAF filter (eg 1–2%) and using a more lenient genotype probability threshold (eg 0.8), or the genotype likelihoods themselves, if missing data are an issue.

### Downstream analyses

For calling runs of homozygosity (ROH), there were subtle differences between the two imputation approaches. Past studies show that imputation with a reference panel can infer ROH in agreement with high coverage data (Sousa da Mota et al. 2023; Bougiouri et al. 2025; Erven et al. 2024). We further show that regardless of whether a reference panel was present or absent, regions inferred as ROH were similar, although imputation without a reference panel inferred a larger proportion of young ROH segments. This behavior could be due to differences between the two datasets, such as the estimated allele frequencies in the 51 samples due to misimputed alleles or missing data. Another difference might be a more extensive set of variants considered in the absence of a reference panel, where variant discovery was performed in 2,800 samples, contrary to the reference panel of 502 samples. However, the strong agreement in the overall ROH classes shows that both approaches can be used to this end. Some variation in the fine scale (length of ROH segment, age of segment) might be expected due to methodological or dataset differences between imputation methods, and care should be taken when making age-specific interpretations. As a consequence, conclusions reached on age and distribution of ROH might not be immediately transferable across studies where different imputation methods have been applied.

In a polygenic trait GWAS, reference-free imputation performed similarly to reference-based. In the GWAS for tarsus length, estimated effects were highly correlated between the two methods. In general, a dataset imputed with an imputation accuracy $r^{2}$ on *N* samples will have equal power as a dataset with $r^{2}\times N$ samples where all genotypes are known (Das et al. 2018). Given the sample size employed (*n* = 1,980) and the similar performance in imputation accuracy in the presence (*r*^2^ = 0.978) or absence (*r*^2^ = 0.97) of a reference panel, we expected the GWAS to have a power of 1,936 and 1,920 samples, respectively. Thus, the high concordance in the effect sizes and *P*-values inferred is not too surprising. This supports that both reference-free and reference-based imputations can be suitable for a GWAS, a goal they are often used for (Hooper et al. 2024; Justen et al. 2024). However, we caution that findings will depend on trait architecture, sample size employed, and species diversity. In summary, researchers should carefully design their experiments so that they have enough power to detect effects in their specific system. Both imputation approaches can be used to that end (Figure S21).

### Implications and perspectives

Many aspects of imputation in wild datasets remain to be explored as genomic imputation gains traction in non-model systems. The reality might be that every investigation will be served by different combinations of underlying genetic diversity, sample relatedness, attainable reference panel size and affordable sequencing depth. Presently, there is a dearth of studies providing benchmarks of imputation accuracy in the different regions of the parameter space and assessing the impact in relevant downstream analyses. While many case studies in different species can reveal the limitations and benefits of different imputation approaches under different realistic conditions, an additional valuable resource might be the development of versatile and easy to implement benchmarking tools. For example, publicly available catalogs of species’ demographic and genetic parameters (Adrion et al. 2020; Gower et al. 2025) coupled with powerful simulation software (Kelleher et al. 2016) allow the rapid generation of large datasets. Such resources coupled with much needed, accessible imputation pipelines (Das et al. 2016) to quickly and reproducibly implement genomic imputation will allow researchers to test imputation performance under a feasible parameter space of reference panel sizes, lcWGS sample sizes, demographies, and genetic diversity (Vi et al. 2025). Benchmarking in real and simulated data can supplement each other, and both will be useful in providing accurate guidelines for sound implementation of imputation in wild systems.

**Figure 6:**
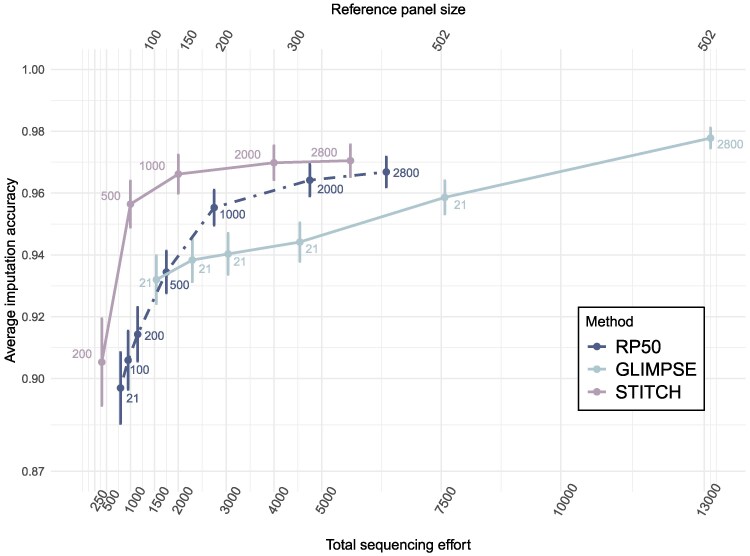
Comparison of imputation accuracy as a function of total sequencing effort. The imputation accuracy for all datasets in this study. Bottom *x*-axis is total sequence effort defined as 2 times the lcWGS size + 15 times reference panel size, with multipliers corresponding to average sequencing depth in each dataset. Top *x*-axis shows the reference panel size, shown when imputing the 21 lcWGS with GLIMPSE. RP50 refers to a reference panel of 50 samples. Text by points shows the 2× lcWGS sample size imputed. Mean and 95% confidence intervals are estimated from the 21 replicate individuals.

An understanding of the strengths and limitations of different imputation strategies can help balance a fixed budget with the objectives of a study. Using our results, we can test the imputation quality of different combinations of sample sizes in the absence or presence of a reference panel (Fig. 6). Imputation with a large well-phased reference panel is the golden standard but might be unattainable in many systems. As an alternative, imputation of 500 or more lcWGS at a sequencing depth of 2×, outperforms imputation with a small reference panel (here 50 samples). While not explicitly tested in our study, we can reasonably assume that larger reference panels (say upwards of 100 samples) will outperform a reference-free approach for the same lcWGS size. However, a reference panel will be limited to the diversity observed in its samples while a large collection of lcWGS samples will allow discovery of a larger set of variants. Therefore, the construction of small reference panels needs to be weighed against the benefit of including more samples in the low-coverage dataset. Our results serve to illustrate that lcWGS without a reference panel has substantial potential in the generation of large datasets in non-model species. We hope that careful benchmarking, along with accurate imputation methods and increasingly affordable DNA sequencing will usher wild populations into the era of large genomic datasets.

## Methods

### Reference panel

The set of individuals used as a reference panel were initially sequenced in multiple past studies (Cumer et al. 2022a, 2022b, 2024; Machado et al. 2022a, 2022b; Topaloudis et al. 2025). Whole-genome sequences of 502 individuals were genotyped together through a published variant discovery pipeline briefly described below (for details, see Topaloudis et al. 2025). Raw reads were processed with trimmomatic v0.39 (Bolger et al. 2014) removing sequencing adapters, excluding unpaired reads and excluding reads smaller than 70 bp. Mapping was performed with BWA-MEM v0.7.17 (Li and Durbin 2009) on the barn owl genome assembly v4 (Machado et al. 2022a). Variant discovery followed the GATK v4.2.6 (Auwera et al., 2013) pipeline with a base quality score recalibration (BQSR) step performed using a previously published “truth set” of variation (Cumer et al. 2022a). GATK's *HaplotypeCaller* ran with default parameters for each individual followed by a common joint variant calling using *GenotypeGVCFs*. SNPs were then filtered to be bi-allelic, pass technical filters (QD < 2.0, QUAL < 30, SOR > 3.0, FS > 60.0, MQ < 40.0, MQRankSum < −12.5, and ReadPosRankSum < −8.0) and to exclude regions of the genome where the mappability is limited (see Corval et al. 2025 for details). Further, genotypes were filtered on individual depth using *BCFtools* v.1.15.1 (Danecek et al. 2011). Any genotype call with a read depth lower than 5 or higher than three standard deviations above the average individual depth was set to missing. After these filters, the reference panel consisted of 10,451,268 SNPs. For this study, we only retained variants present on the 39 autosomal linkage groups of the barn owl genome (Topaloudis et al. 2025), resulting in a total of 10,115,035 autosomal SNPs.

The reference panel was phased in two steps. First, individual variants were pre-phased using an observational approach in which reads or pairs of reads covering multiple heterozygous sites were used to resolve local haplotypes (read-based phasing). Individuals coming in trios (both parents and an offspring), were phased using trio information (genetic phasing). Both phasing approaches were coupled using *WhatsHap* v1.4 (Garg et al. 2016; Martin et al. 2016) with the mean recombination rate estimated for the species (–*recombrate* 2) and individuals belonging to one (or more) trio were phased using *WhatsHap's pedigree-mode*. Offspring were phased with their parents, and parents which had more than one offspring were phased along with the highest sequencing depth offspring.

After read-based and genetic phasing, we performed an additional step of filtering. We identified 187 unrelated individuals (kinship <0.03125) and retained bi-allelic SNPs with minor allele count larger than five (MAC > 5) and missing data <5% based on this set of unrelated individuals to account for non-independence of sampling in the family data.

This set of filtered variants was then statistically phased with SHAPEIT v4.1.2 (Delaneau et al. 2019). We phased the 187 unrelated individuals and 315 individuals belonging to families separately to avoid effects of family structure in the dataset. First, the 187 unrelated individuals were phased together and then each family member was independently phased with the unrelated set. We ran SHAPEIT with the number of conditioning neighbors in the PBWT set to 8, and the MCMC chain running with ten burn-in generations, five pruning iterations, each separated by one burn-in iteration, and ten main iterations.

The quality of the phasing was assessed using the switch error-rate (Browning and Browning 2011) metric: when comparing two phase sets for an individual's variants, a switch-error occurs when a heterozygous site has its phase switched between the phase sets. For each individual, the local phasing inferred from WhatsHap (read-based approach and when available genetic approach) was considered as the ground truth. This phase set was compared with the statistical phasing of this individual using Shapeit, with read-based/genetic phase information ignored for the individual. The final estimation of the switch error rate was done using the switchError code to compare both phasing sets (available at https://github.com/SPG-group/switchError). The results showed that the phasing with SHAPEIT yielded a low error rate (mean error rate of 1.83%, Cumer et al. 2025).

### Low coverage dataset

We sequenced 2,800 owls from a pedigreed population in Switzerland (Roulin et al. 1998; Frey et al. 2011). Blood samples were collected between 1986 and 2020 and individuals with complete family information and phenotypic data available were prioritized for sequencing. For verification of sequencing performance, we included 32 individuals that had already been sequenced at high coverage in the reference panel. These individuals sequenced at both high and low coverage were used to measure the accuracy of imputation strategies.

For the lcWGS, genomic DNA was extracted from blood samples stored at −80 °C using the DNeasy Tissue and Blood Kit (Qiagen, Switzerland) on columns or with the Biosprint robot 96 (Qiagen, Switzerland) and stored at −20 °C. The DNA was quantified using Quant-it PicoGreen dsDNA Assay kit (Thermo Scientific, Switzerland) and was diluted in 10 mM Tris-HCl to 1.5 to 2.5 ng/μ for library preparation. We randomized the position of the DNA samples of the 2,800 owls on 30 different 96-well plates (to avoid an effect of extraction years and DNA quality on the library preparation and sequencing). Every plate included two wells containing Tris buffer only to measure potential contamination during Library preparation and sequencing. The libraries were prepared with 8 plexWell low-pass (LP) 384 kits (SeqWell, USA) and sequenced in three flow cells of an Illumina NovaSeq 6000 at the genomic technologies facility of the University of Lausanne (GTF).

Raw paired-end 150 bp reads were trimmed with *Trimmomatic* v.0.36 (Bolger et al. 2014) and aligned to the barn owl reference genome v4 (Machado et al. 2022a) using *BWA-MEM* v.0.7.15 (Li and Durbin 2009). Mean coverage of the genome per individual was 1.95× with a minimum coverage of 0.2× and maximum coverage of 4.15× (Fig. 1c).

### Imputation

#### Imputation using a reference panel

Imputation using GLIMPSE is described in more detail in Cumer et al. (2025). Briefly, we determined the genotype likelihood of each lcWGS sample at each variant position present in the reference panel using *BCFtools* v.1.15.1 (Danecek et al. 2021) *mpileup* and *call* methods, using the -T and -C options. We then used *GLIMPSE* v1.1.1 (Rubinacci et al. 2021) for the imputation and phasing of the low coverage individuals using the reference panel described above. We used the first version of GLIMPSE (v1.1.1) because it is more suitable for small reference panels. The first step of the GLIMPSE pipeline, called chunking (*GLIMPSE_chunk*), splits the chromosome into chunks for efficient imputation and phasing. Chunking was performed with default parameters. The second step consists of the phasing and imputation of the genomic chunks (*GLIMPSE_phase*). This method iteratively improves low-coverage genotypes’ likelihoods and phasing for each individual independently. The *GLIMPSE_phase* method was used with the maximum number of iterations (–*burnin* 100 and –*main* 15) and with a large effective population size parameter value (–*ne* 10,000). The family-based recombination map of the species (Topaloudis et al. 2025) was included with the –*map* parameter. The third step is the ligation step, which merges the different chunks without losing phase information (*GLIMPSE_ligate*) and was executed with default parameters. The last step of the *GLIMPSE* pipeline is haplotype identification (*GLIMPE_sample*) which identifies the most likely haplotype based on posterior genotypes’ likelihood and phase information (we ran with the –*solve* mode). Replicate samples were imputed by removing their high-coverage genotypes from the reference panel.

For subsets of the reference panel, the 22 replicates (21 high coverage and one sample which was not used for benchmarking) were imputed with a random selection of 50,100,150,200,300 samples from Switzerland, and with the full reference panel (502–22 = 480 samples). Individuals were chosen by prioritizing founders in the pedigree and individuals with multiple offspring. Phasing was rerun using WhatsHap in read-based (not trio) mode and SHAPEIT. All other parameters were the same as in the full run. For a reference panel size of 50, we also imputed different sizes of lcWGS samples. The sizes were 100; 200; 500; 1,000; 2,000 and the full dataset of 2,800 lcWGS.

We also imputed the 2,800 lcWGS using the full reference panel of 481 samples (502–21) using QUILT v2.0.4 (Li et al. 2025). We used the first version of QUILT (Davies et al. 2021) which is better suited for small reference panels. We used the same sites as in GLIMPSE. We created the haplotype legends sample files using *bcftools convert* and ran *QUILT_prepare_reference.R* script for a 5 Mb region with 1Mb buffer, using 1350 as nGen. Then we ran QUILT.R using the same parameters.

#### Imputation without a reference panel

STITCH v1.7.2 (Davies et al. 2016) was used to impute the genotypes of the 2,800 low coverage samples. The software models the population being founded by *K* founders *n* generations ago, and requires a list of positions to genotype in all samples. In all runs, *n* can be defined as $\frac{4\times Ne}{K}\;\;$, with *Ne* being the effective population size (in our case, we used 10,000 in line with the GLIMPSE implementation). The optimal number of *K* is case-dependent and needs to be determined. We chose two linkage groups with different recombination landscapes, one with a punctuated landscape of recombination (LG22, 165 k SNPs, 25 Mb in length, ∼2.5  cM/Mb) and one with elevated recombination across its length (LG36; 80 k SNPs, 7.8 Mb in length, ∼6.4 cM/Mb) (Topaloudis et al. 2025). We genotyped SNP positions present in the reference panel and ran STITCH using the “diploid” method on the full dataset of 2,800 samples for values of *K* ranging from 4 to 40 with a step of 2. We then compared the squared correlation of the inferred genotypes with the “true” genotypes for the 32 replicate individuals to identify an optimal value of *K* (Figure S22). We chose *K* = 30 as a tradeoff of running time and accuracy which we used for all downstream runs of STITCH.

To run STITCH a set of variant positions is needed. In order to follow a similar pipeline as a study would, we decided to perform de-novo snp-calling in the lcWGS dataset. We did this using *BCFtools* v.1.15.1. The genotypes were generated with the *bcftools mpileup* command with the following parameters (-B, -q 20 -Q 20 -a “INFO/AD,AD,DP”) using the barn owl reference genome v4 and the mappability mask mentioned above (see Reference Panel). Then variant positions were called using the *bcftools call* command with calling indels (-V indels) on a multivariate caller mode (-mv). Variants were filtered for missingness (maximum 30%), genotype quality (minimum 20) and total depth (minimum 1,000) and turned into a position file for use in STITCH using *bcftools query*. Then, STITCH was run in 5Mb windows setting K to 30, and the number of generations to 1,350 with the method set to the diploid model for 40 iterations and all other parameters as default. After imputing all sites, we filtered out any site with an INFO_SCORE < 0.4 and a Hardy–Weinberg equilibrium *P*-value < 1e−6 and rerun STITCH on this subset of SNPs. This time we included the parameters –*niterations* = 60 –*shuffleHaplotypeIterations* = “c(4,8,12,16,20,24)”.

To test for the effect of sample size, we subsampled the low coverage datasets to sizes of 50; 100; 200; 500; 1,000, and 2,000. We always included the 32 replicated individuals and the rest of the individuals were picked by either random sampling or by sampling individuals prioritizing families. Families were defined by sharing a family ID generated with the *makefamid* function in the R package *kinship2* v1.9.6 (Sinnwell et al. 2014). For each subset, we always chose the largest possible family to include, and repeated the process until the subset was filled.

We also imputed the lcWGS dataset without a reference panel using a combination of ANGSD, for SNP calling (Korneliussen et al. 2014) and BEAGLE, for imputation (Browning and Browning 2009) as described in the ANGSD manual. Because STITCH outperformed ANGSD/BEAGLE, we only present the results for STITCH in the main text but the results for BEAGLE can be found in the Supplementary material.

### Downstream comparison

#### Statistics

We used the 32 replicates to quantify imputation accuracy. Of those one was a library error and excluded from all analyses. Ten of these samples were sequenced in intermediate depth in the “truth” dataset (mean depth 11×; range 8.4–17.3×; hereafter “intermediate-depth replicates”) while 21 were sequenced in high depth (mean depth = 30.5, range = 22.7–40.8, hereafter “high-depth replicates”).

The per sample accuracy of imputation, *r*^2^, is the squared correlation of the genotype vectors for each individual between the truth (high coverage) and the imputed dataset. We also estimated the per sample imputation accuracy on the centered and scaled genotype vectors as suggested in Calus et al. (2014). The scaled imputation accuracy gives larger weight to rare alleles and results were identical in rank but smaller in absolute value when using the scaled correlation (Figure S23). In all comparisons, the reference allele was chosen to be the most frequent allele across all methods. We estimated sensitivity (true positive rate) the ratio of true positives to the sum of true positives and false negatives and precision (positive predictive value) the ratio of true positives to the sum of true positives and false positives. All statistics were estimated from comparing the genotype matrices generated either from GLIMPSE or STITCH or by extracting the information from the *vcf* files. Since GLIMPSE does not report missing genotypes, we converted the *vcf* file into dosage format. For genotypes set to missing in STITCH, we extracted the genotype dosage from the *GT:DOS vcf* field as reported by STITCH. For STITCH, float genotype values from likelihood calls were rounded to the nearest integer (lenient threshold) for comparison with the integer high coverage genotypes and all statistics were estimated using the *confusionMatrix* function of the R package *caret* v.6.0-94 (Kuhn and Max 2008). All imputation software output an accuracy metric per variant reflecting the confidence in the imputed genotypes. The metric used in GLIMPSE and STITCH is the imputation “information score” (INFO score). All individual accuracy measures were estimated for three INFO cutoff filters (0, 0.6, and 0.8) and with or without taking into account the missing genotypes in STITCH.

To identify the source of variation in per sample imputation accuracy, within each method, we regressed the per sample imputation accuracy of each high-depth replicate to the relatedness in each dataset (reference panel and lcWGS dataset; see Figures S19 and S20). We also tested the effect of the sequencing depth in high or low coverage, using the *lm* function in R.4.4.1. Relatedness was calculated using a doubled kinship matrix of the samples estimated with *hierfstat* v.0.5-11 (Goudet 2005). A PCoA of all our samples was performed on a distance matrix estimated from a relatedness matrix using the *pcoa* function of R package *ape* v5.8-1.

To quantify per variant imputation accuracy, we compared the genotype vectors of the high-depth replicates. To quantify imputation accuracy in rare alleles, we used the non-reference-concordance (NRC). We define NRC as $NRC\;=\;1\;-\frac{\left(e_{RR}+e_{RA}+e_{AA}\;\right)}{\;(e_{RR}+e_{RA}+e_{AA}+\;m_{RA}+m_{AA}\;)}\;$, where $e_{x}$ refers to the number of mismatches of genotypic class “x” and $m_{X}$ refers to the number of matches in genotypic class “x.” Genotypic classes are defined as RR: homozygous for the common allele, RA: heterozygotes, AA: homozygous for the rare allele. We quantified NRC for each set of variants in the following allele frequency bins “0.00001 0.00100 0.00200 0.0030 0.0040 0.00500 0.01000 0.01500 0.02000 0.02500 0.03000 0.04000 0.05000 0.10000 0.20000 0.30000 0.40000 0.50000” using the *GLIMPSE_concordance* tool of GLIMPSE v1.1.1. The tool was run using the genotype mode *(–gt-validation, –gt-target*) for each imputed dataset. We quantified allele frequencies in four datasets: 76 unrelated Swiss samples present in the reference panel, the whole reference panel (*n* = 502), the imputed dataset from GLIMPSE (*n* = 2,800) and the imputed dataset from STITCH (*n* = 2,800). Allele frequencies were highly correlated and most accurate in large sample sizes as expected and for the comparison of NRC per MAF bin we used the allele frequencies estimated in GLIMPSE (Figure S24).

#### Homozygosity-by-descent estimation

To generate estimates of homozygosity-by-descent (HBD) and annotate the origin of HBD segments along the genome we used the *RzooRoH* v0.4.1 R package (Bertrand et al. 2019). A subset of 51 individuals was used (21 high-depth replicates and 30 individuals previously shown to be inbred Hewett et al. 2025). The 30 inbred individuals sequenced on lcWGS were included to quantify the relative performance of imputation in more highly inbred individuals. We firstly filtered for INFO > 0.8 and an allele frequency of 0.1%, then ran the default RzooRoH model with the number of K-rates set to 14, corresponding to HBD origins of 2; 4; 8; 16; 32; 64; 128; 256; 512; 1,024; 2,048; 4,096; and 8,192 generations ago.

#### GWAS

To test the performance of the imputed dataset in a genome wide association study we used the mean tarsus length (mm) of adult birds, a polygenic phenotype according to preliminary analyses, with intermediate heritability in adults (Hewett et al. 2025). A total of 1,980 individuals were included in the GWAS. A linear mixed model was used in the *gaston* R package v.1.5.9 (Perdry et al. 2018) with the function *association.test*. Sex was fitted as a fixed effect, formatted as a factor. In addition an allele-sharing (Weir and Goudet 2017; Goudet et al. 2018) kinship matrix estimated with *hierfstat* v.0.5-11 (Goudet 2005), doubled to correspond to a relatedness matrix, was used as a random effect to control for structure and relatedness in the dataset. For the construction of the kinship matrix, we filtered the SNPs using plink2 (Chang et al. 2015) based on minor allele frequency (1%) and LD (0.2 in a 100 SNP window with a step of 10) filtered dataset. All R-related analyses were executed under R version 4.4.1. Scripts for the analyses can be found in the following GitHub repository: https://github.com/topalw/Imputation_owls and on Zenodo along with intermediate files under 10.5281/zenodo.18377759.

## Supplementary Material

msag094_Supplementary_Data

## Data Availability

Sequence data used in the study from previous publications are available on NCBI under BioProject codes PRJNA700797, PRJNA727915, PRJNA727977, PRJNA774943, PRJNA925445, and PRJNA1172395. Low coverage sequencing data can be found under BioProject code PRJNA1391678, which will be made available upon publication. Scripts available at https://github.com/topalw/Imputation_owls and on Zenodo under 10.5281/zenodo.18377759. along with intermediate data files.
