# Supplementary material for: Benchmarking imputation accuracy in the presence or absence of a reference panel
Source: Mol Biol Evol. 2026 Apr 10;43(4):msag094. doi: 10.1093/molbev/msag094 (PMC13122032; doi:10.1093/molbev/msag094)
Supplement: msag094_Supplementary_Data [file msag094_supplementary_data.pdf]

Supplementary Material

Supplementary Text 1 ..... **2**
Running ANGSD and BEAGLE 2
Downstream 2
Supplementary Tables **3**
Supplementary Figures **4**

Supplementary Text 1

Imputation using BEAGLE

We genotyped individuals sequenced at low coverage using a combination of ANGSD v.0.931 (Korneliussen et al. 2014) for SNP calling followed by Beagle v.3.3.2 (Browning and Browning 2009) for phasing and imputation, following a pipeline similar to that implemented in (Lou et al. 2021).

Genotypes and genotype dosages were first estimated in ANGSD using the following settings: *-GL 2 -doGlf 2 -doMaf 1 -doMajorMinor 4 -doCounts 1 -remove_bads 1 -minMapQ 30 -minQ 20 -skipTriallelic 1 -minInd 1000 -setMaxDepthInd 10 -setMinDepthInd 1 -SNP_pval 1e-6.*

SNP calling was restricted to regions of the genome with high mappability, as described in Corval et al. (2023). Genotype imputation was then performed using Beagle v.3.3.2 with default parameters, using the genotype likelihoods output by ANGSD in Beagle format (.beagle.gz) as input. Imputation was conducted without a reference panel. For the imputed datasets generated by BEAGLE we compared the dosage (rounded to integers) with the dosage of the high coverage dataset of the 21 replicates (Supplementary Figure 25).

References

Browning BL, Browning SR. 2009. A Unified Approach to Genotype Imputation and Haplotype-Phase Inference for Large Data Sets of Trios and Unrelated Individuals. *The American Journal of Human Genetics* 84:210–223.

Korneliussen TS, Albrechtsen A, Nielsen R. 2014. ANGSD: Analysis of Next Generation Sequencing Data. *BMC Bioinformatics* 15:356.

Lou RN, Jacobs A, Wilder AP, Therkildsen NO. 2021. A beginner's guide to low-coverage whole genome sequencing for population genomics. *Molecular Ecology* 30:5966–5993.

Supplementary Tables

Supplementary Table 1. Number of SNPs used in the study. Percentages in parentheses are compared to the non filtered datasets.

Method	Number of SNPs		
	**No filtering**	**0.6**	**0.8**
**GLIMPSE**	10,102,233 (100%)	9,709,936 (96%)	9,526,404 (94%)
**STITCH**	21,732,148 (100%)	21,254,734 (98%)	21,002,173 (97%)
**BEAGLE**	45,746,186 (100%)	17,303,660 (38%)	8,212,527 (18%)

Supplementary Table 2. Number of unique and overlapping SNPs among methods. Method–unique SNPs in the diagonal. Proportion of each method's total (row) in parentheses.

Method	GLIMPSE	STITCH	BEAGLE
**GLIMPSE**	383,175 (4%)	7,868,202 (78%)	9,567,325 (95%)
**STITCH**	7,868,202 (36%)	76,514 (0.4%)	21,626,081 (99%)
**BEAGLE**	9,567,325 (20%)	21,626,081 (47%)	20,712,846 (45%)

Supplementary Figures

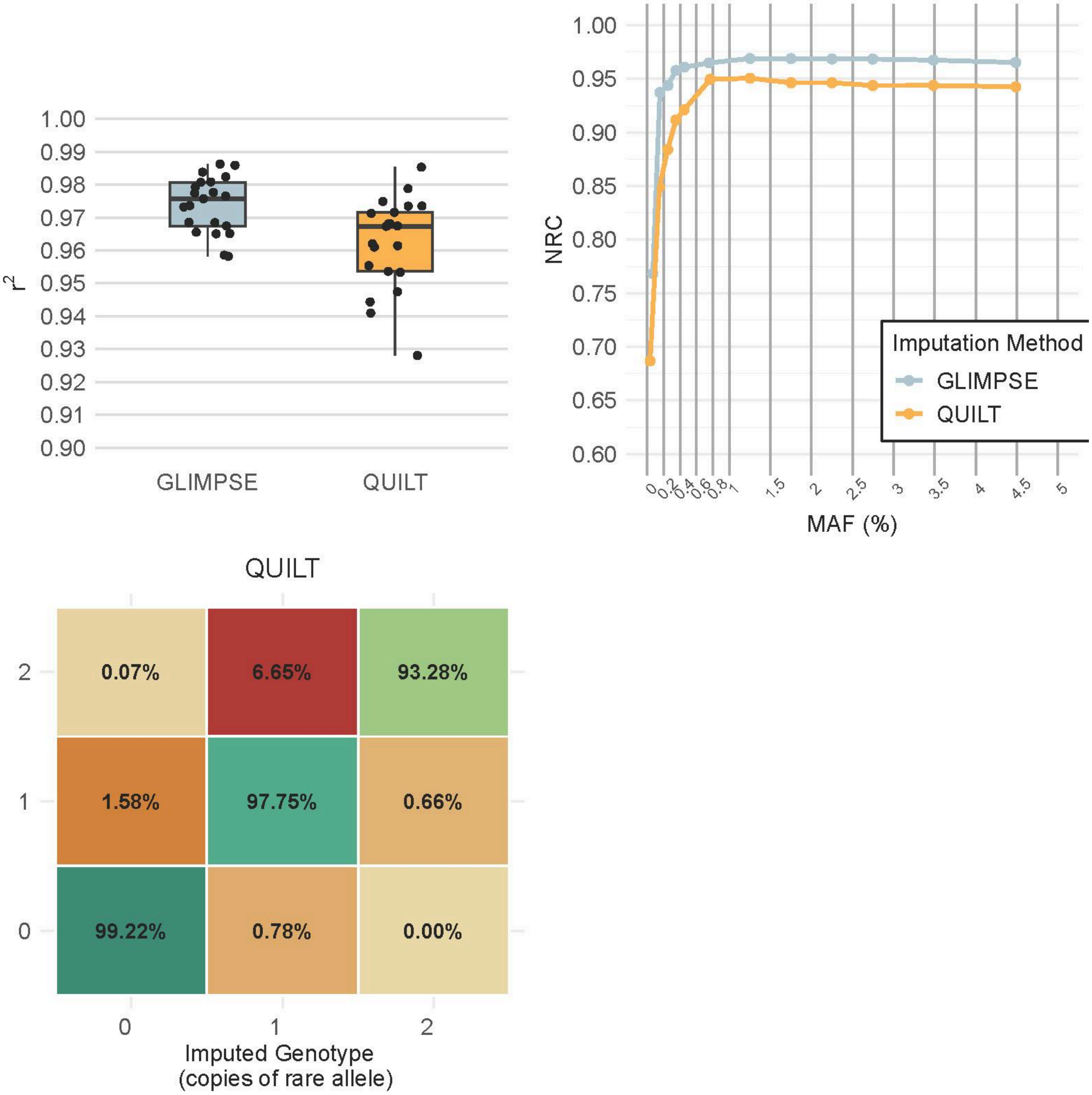

Supplementary Figure 1. The imputation accuracy including the QUILT imputed dataset. A) Per sample imputation accuracy. B) The non-reference concordance (NRC) along different low-frequency alleles (effects estimated per SNP instead of in bins as in main text). C) The overall misclassification matrix when using QUILT.

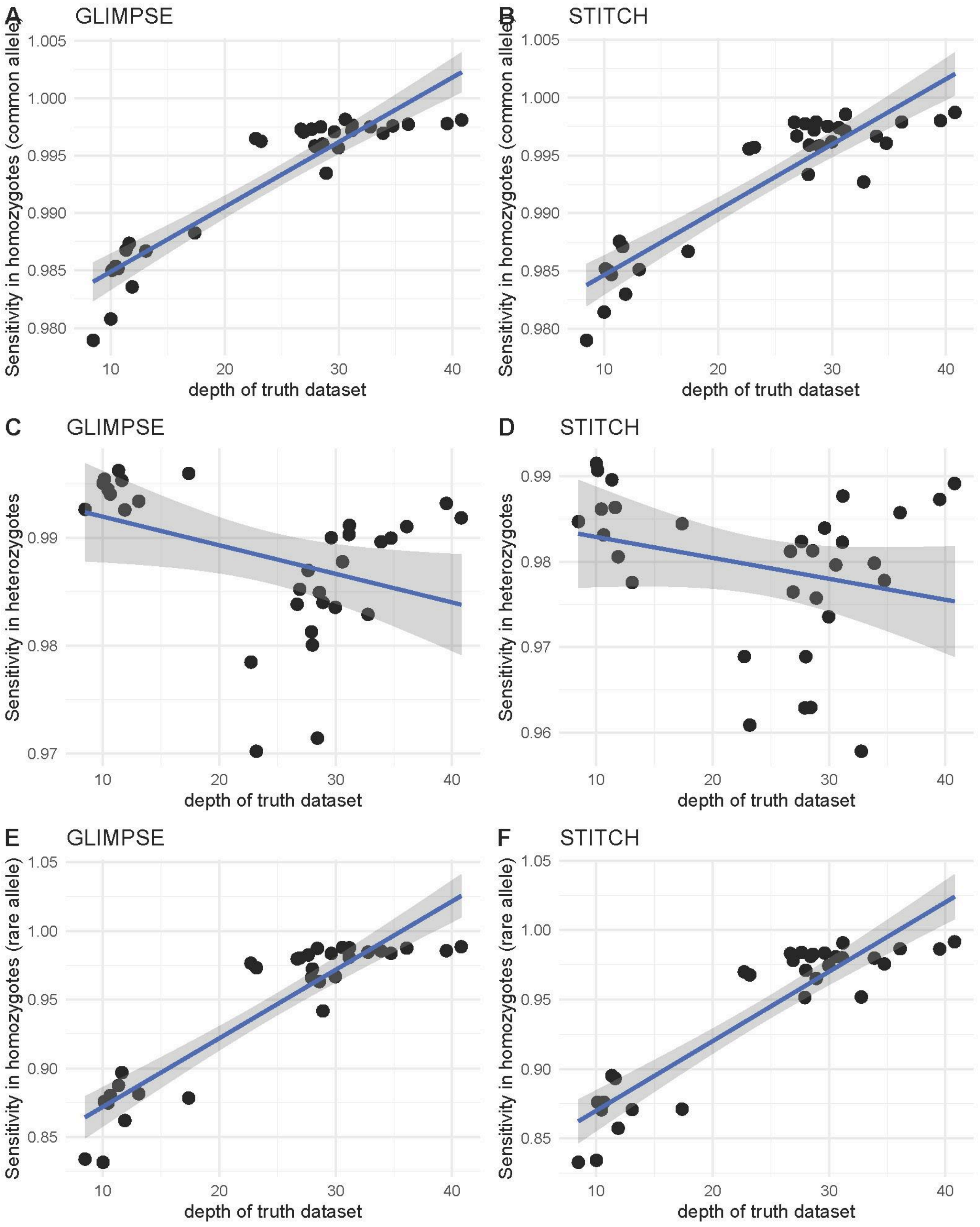

Supplementary Figure 2. Sensitivity of each genotype class (rows), with each method (columns) as a function of the sequencing depth used as the 'truth'.

Sensitivity refers to the ratio of true positives over true positives and false negatives and can be thought of as the proportion of true genotypes in one class (e.g. heterozygotes) inferred by the imputation method. **A,B**) Sensitivity

of homozygotes for the common allele with GLIMPSE (A) and STITCH (B), **C,D**) Sensitivity of heterozygotes with GLIMPSE (C) and STITCH (D), **E,F**) Sensitivity of homozygotes for the rare allele with GLIMPSE (E) and STITCH (F). Note the different pattern among heterozygotes and homozygotes.

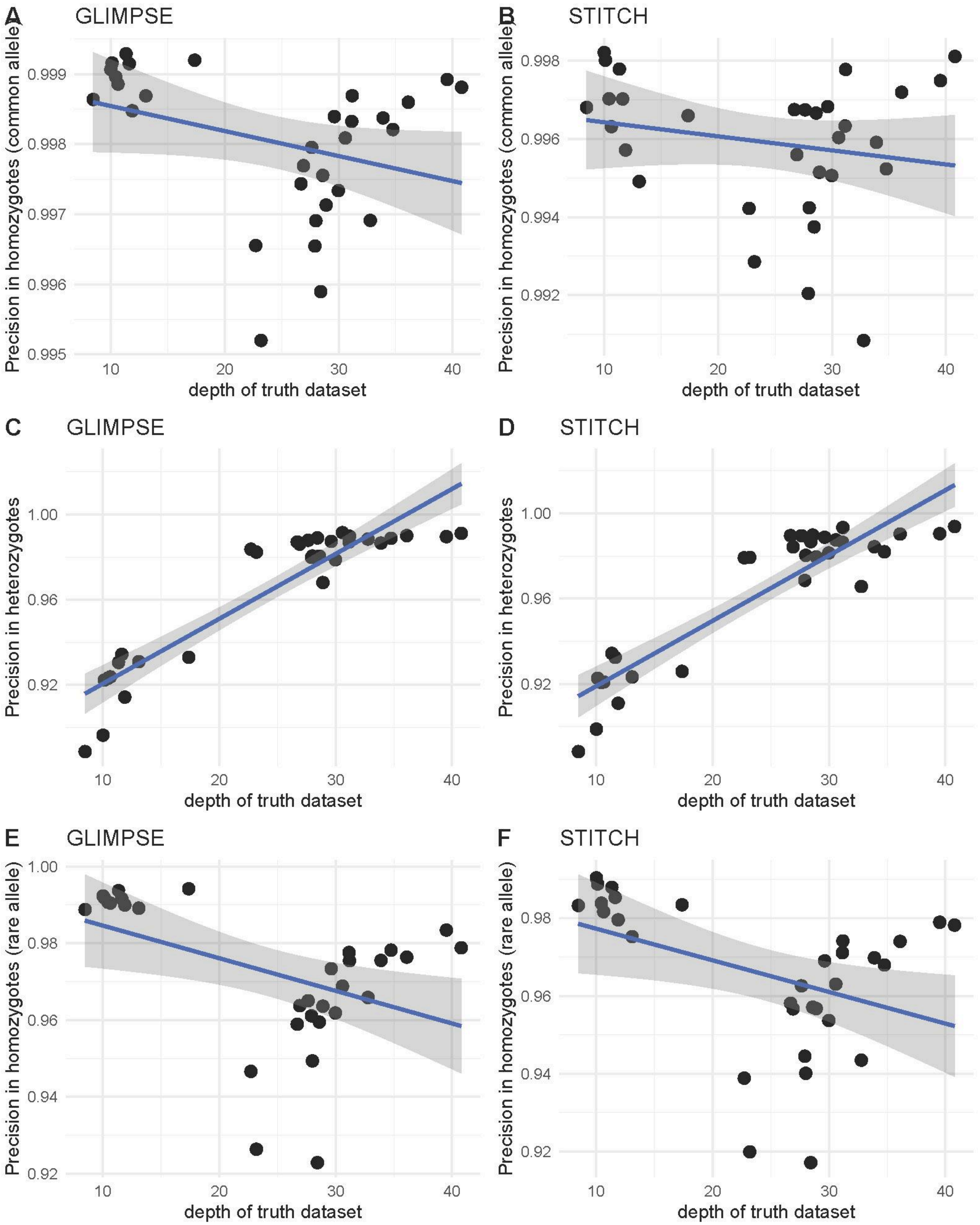

Supplementary Figure 3. Precision of each genotype class (rows), with each method (columns) as a function of the sequencing depth used as the 'truth'.

Precision refers to the ratio of true positives over true positives and false positives and can be thought of as the proportion of genotypes imputed in one class (e.g. heterozygotes) truly being in that class. **A,B**) Sensitivity of

homozygotes for the common allele with GLIMPSE (A) and STITCH (B), **C,D**) Sensitivity of heterozygotes with GLIMPSE (C) and STITCH (D), **E,F**) Sensitivity of homozygotes for the rare allele with GLIMPSE (E) and STITCH (F). Note the different pattern among heterozygotes and either homozygotes.

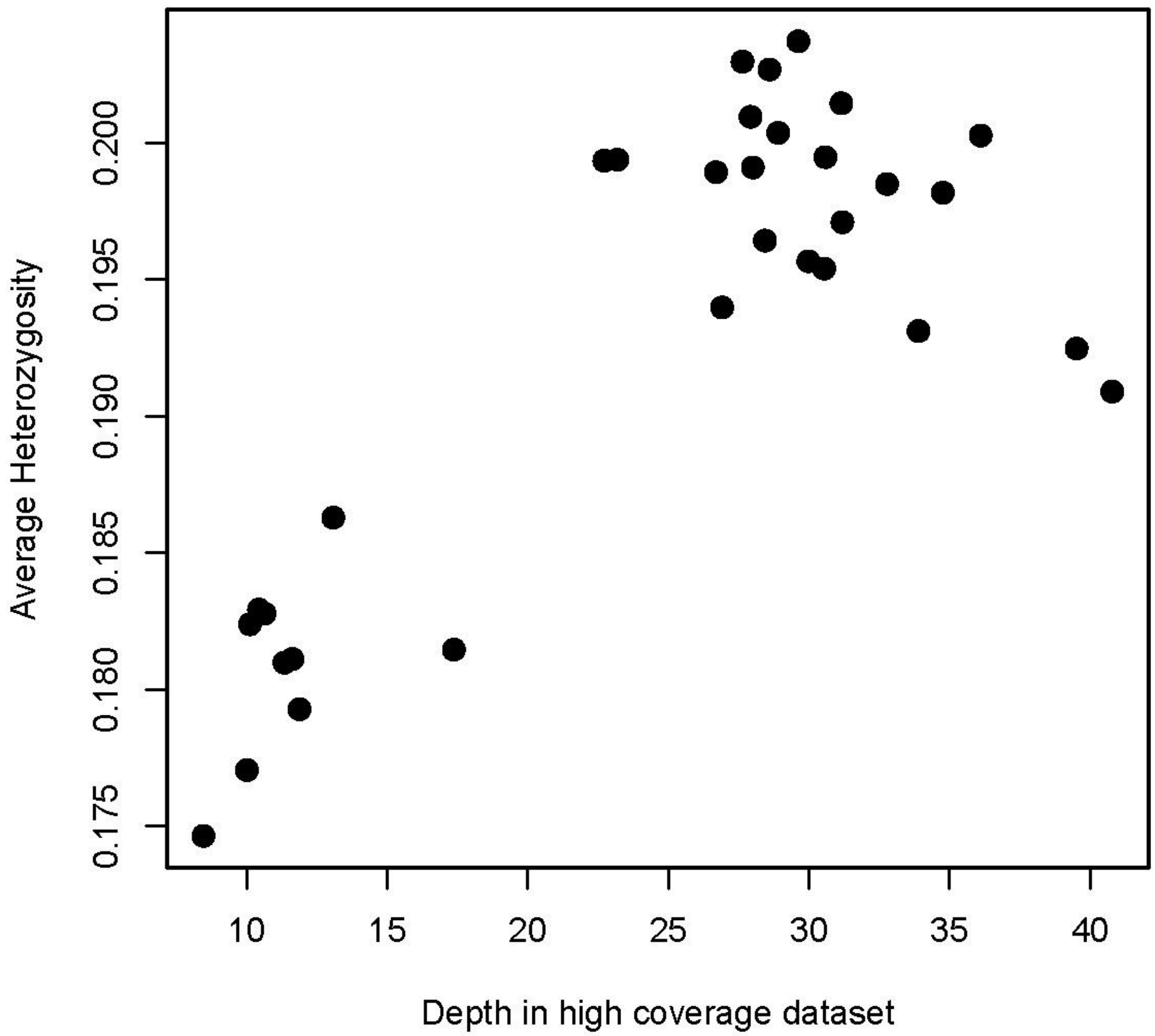

Supplementary Figure 4. The effect of sequencing depth on heterozygosity.

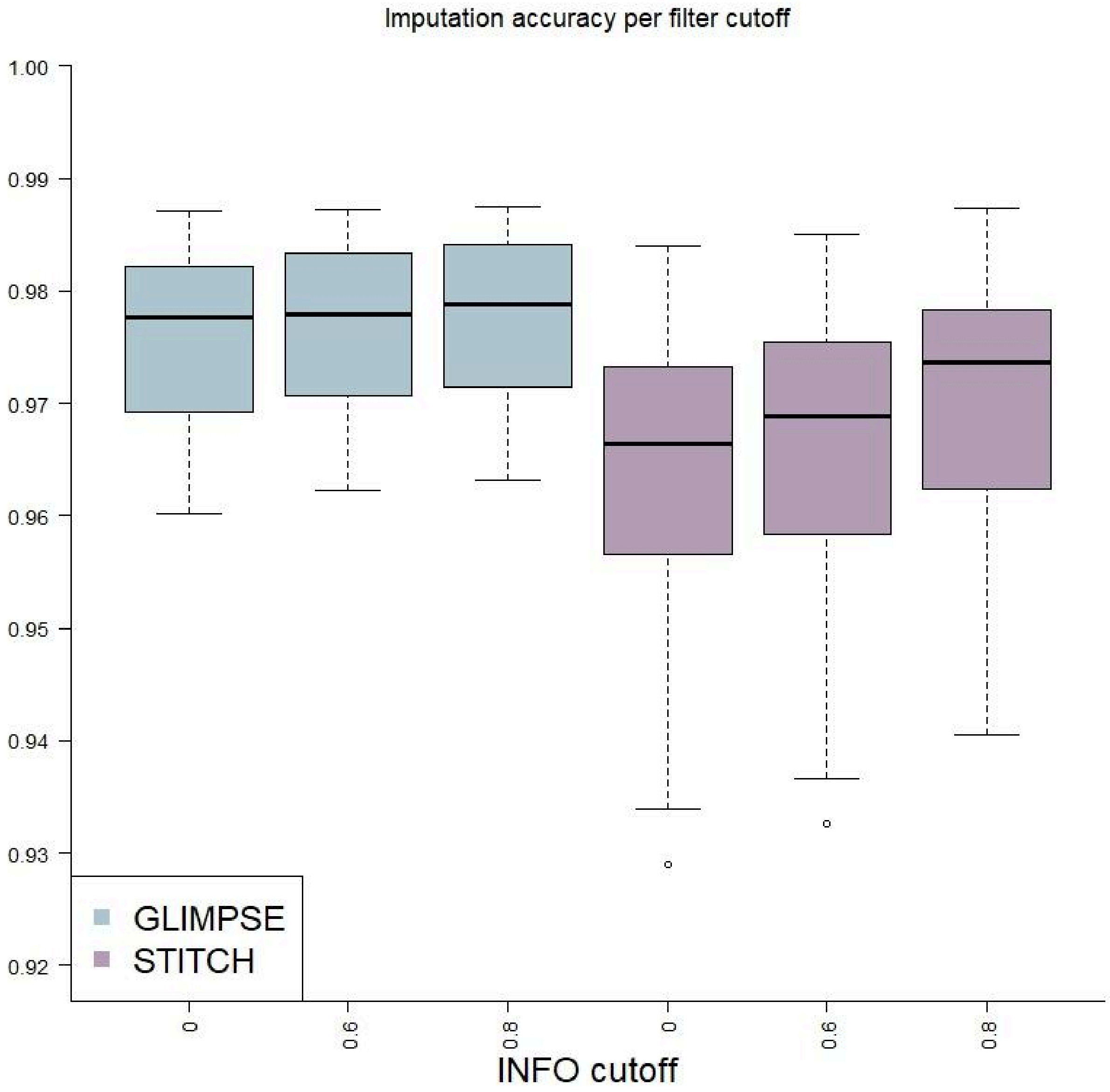

Supplementary Figure 5 The effect of filtering cutoff on imputation accuracy.

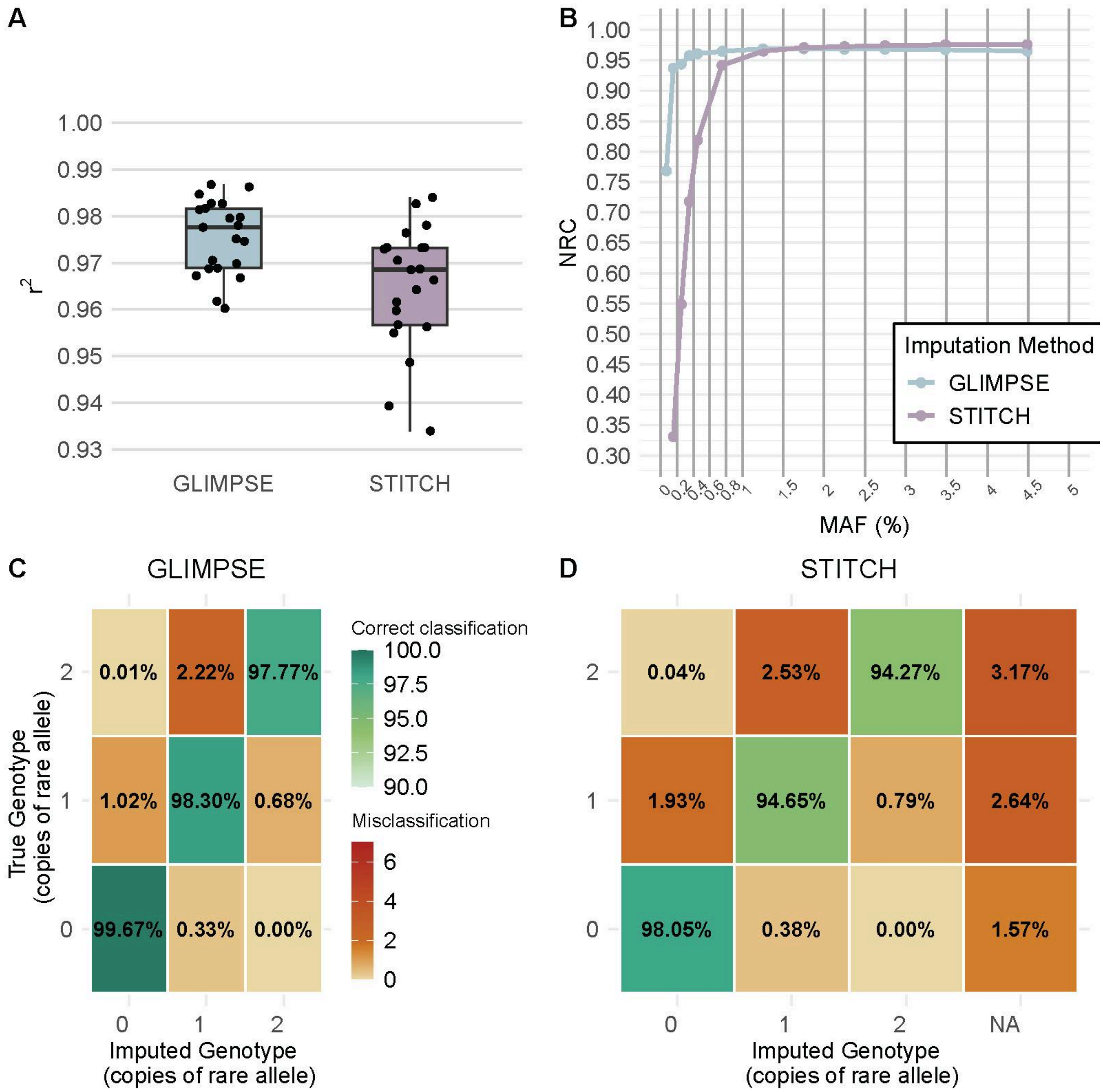

Supplementary Figure 6 - The accuracy of imputation with no INFO cutoff.

A) the squared correlation (imputation accuracy) per sample between the 'truth' (high-coverage data) and the dataset imputed by GLIMPSE or STITCH. Each point is one of the 21 replicate individuals**. B)** Non-reference concordance (NRC) per minor allele frequency bin. Each point represents the mean in the specific allele frequency bin. Allele frequency as defined in 76 unrelated individuals sequenced in high coverage. **C)** Confusion matrix for genotype classification for GLIMPSE. Each row represents the true genotype for these markers and the column the imputed one. **D)** Confusion matrix for genotype classification for STITCH. Each row represents the true genotype for these markers and the column the imputed one. In D the NA column represents uncalled genotypes in STITCH. For C and D, diagonals are colored differently since higher is better, than off-diagonal where lower is better. All statistics calculated along the whole autosomal genome

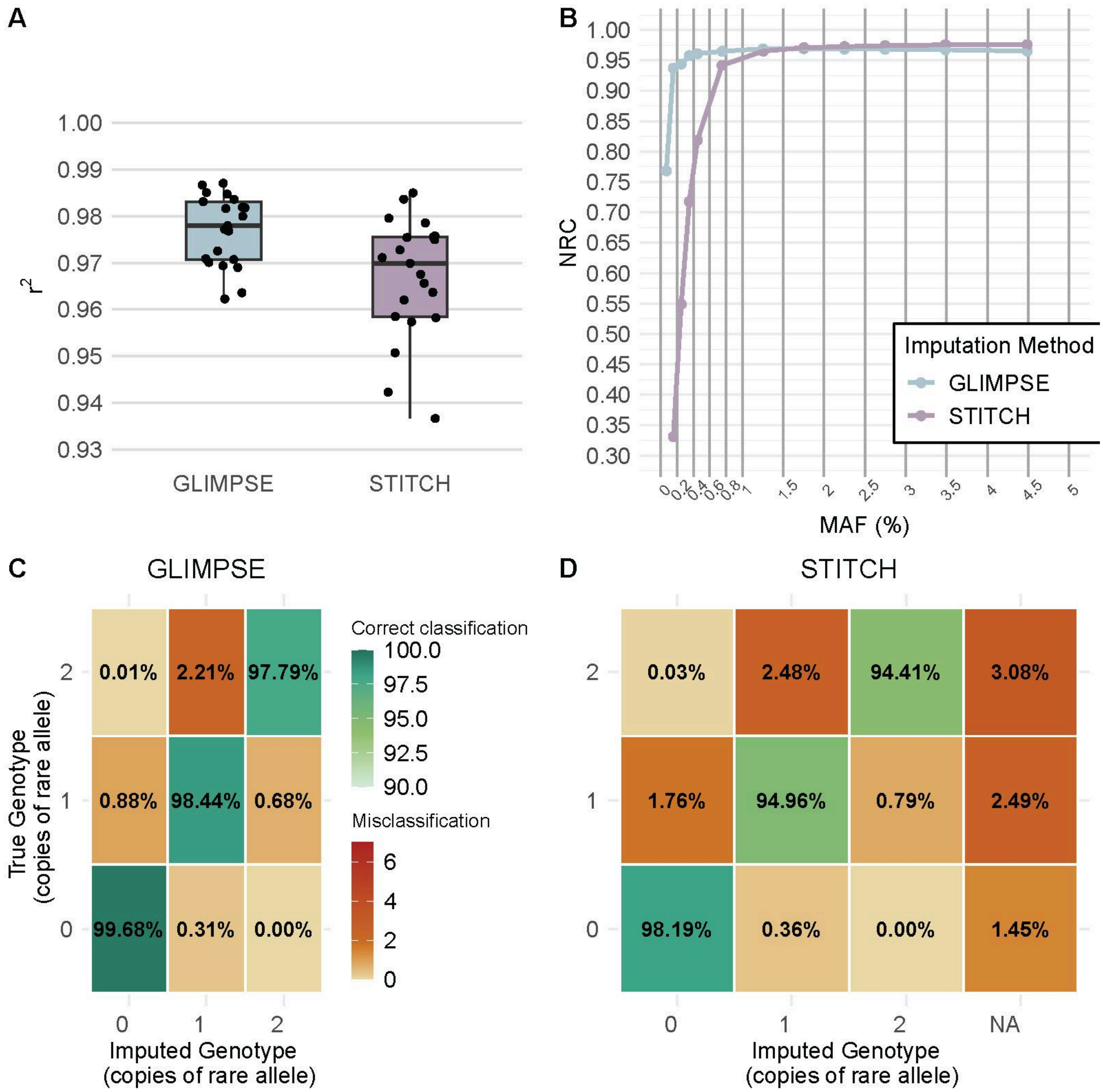

Supplementary Figure 7 - The accuracy of imputation with an INFO cutoff of 0.6.

A) the squared correlation (imputation accuracy) per sample between the 'truth' (high-coverage data) and the dataset imputed by GLIMPSE or STITCH. Each point is one of the 21 replicate individuals**. B)** Non-reference concordance (NRC) per minor allele frequency bin. Each point represents the mean in the specific allele frequency bin. Allele frequency as defined in 76 unrelated individuals sequenced in high coverage. **C)** Confusion matrix for genotype classification for GLIMPSE. Each row represents the true genotype for these markers and the column the imputed one. **D)** Confusion matrix for genotype classification for STITCH. Each row represents the true genotype for these markers and the column the imputed one. In D the NA column represents uncalled genotypes in STITCH. For C and D, diagonals are colored differently since higher is better, than off-diagonal where lower is better. All statistics calculated along the whole autosomal genome.

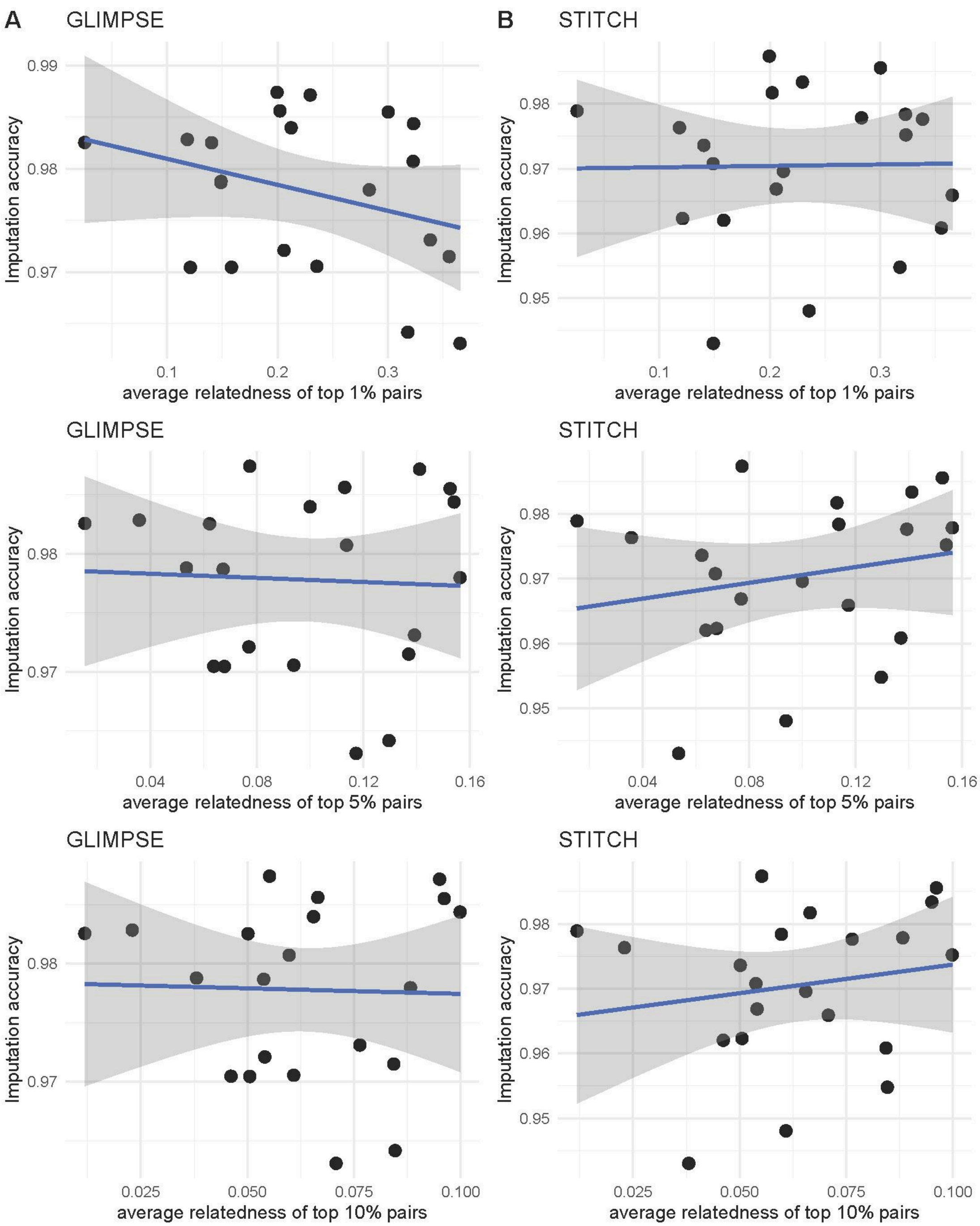

Supplementary Figure 8. Imputation accuracy of the 21 replicates as a function of the degree of relatedness to the reference panel.

Imputation accuracy of each method (columns) as a function of the average relatedness of the top 1%, 5% and 10% of relatives in the reference panel. No relationship is significant.

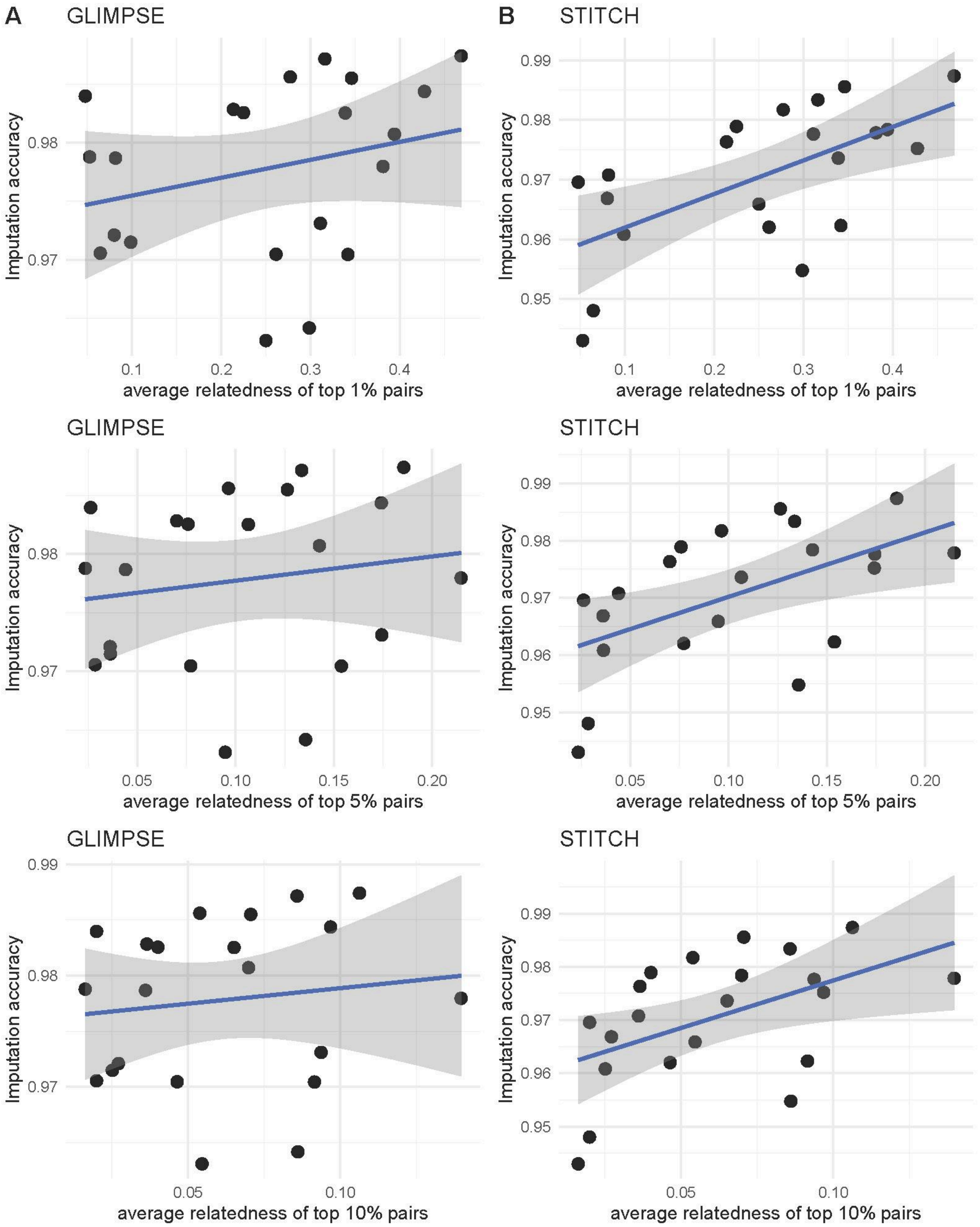

Supplementary Figure 9. Imputation accuracy of the 21 replicates as a function of the degree of relatedness to the lcWGS dataset.

Imputation accuracy of each method (columns) as a function of the average relatedness of the top 1%, 5% and 10% of relatives in the lcWGS dataset. All slopes in the right column (STITCH) are significantly different from 0.

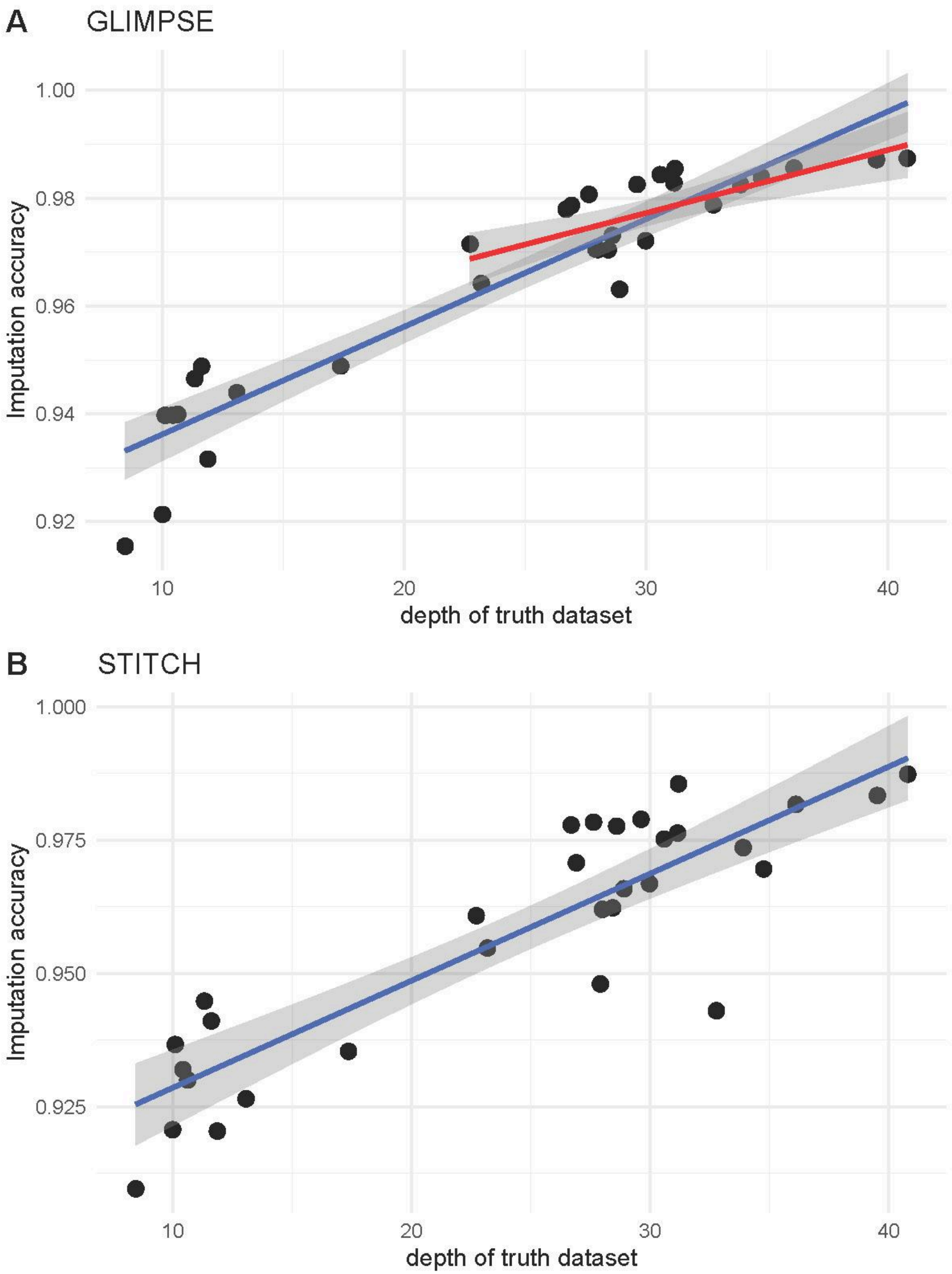

Supplementary Figure 10 - Imputation accuracy as a function of the 'truth' sequencing depth.

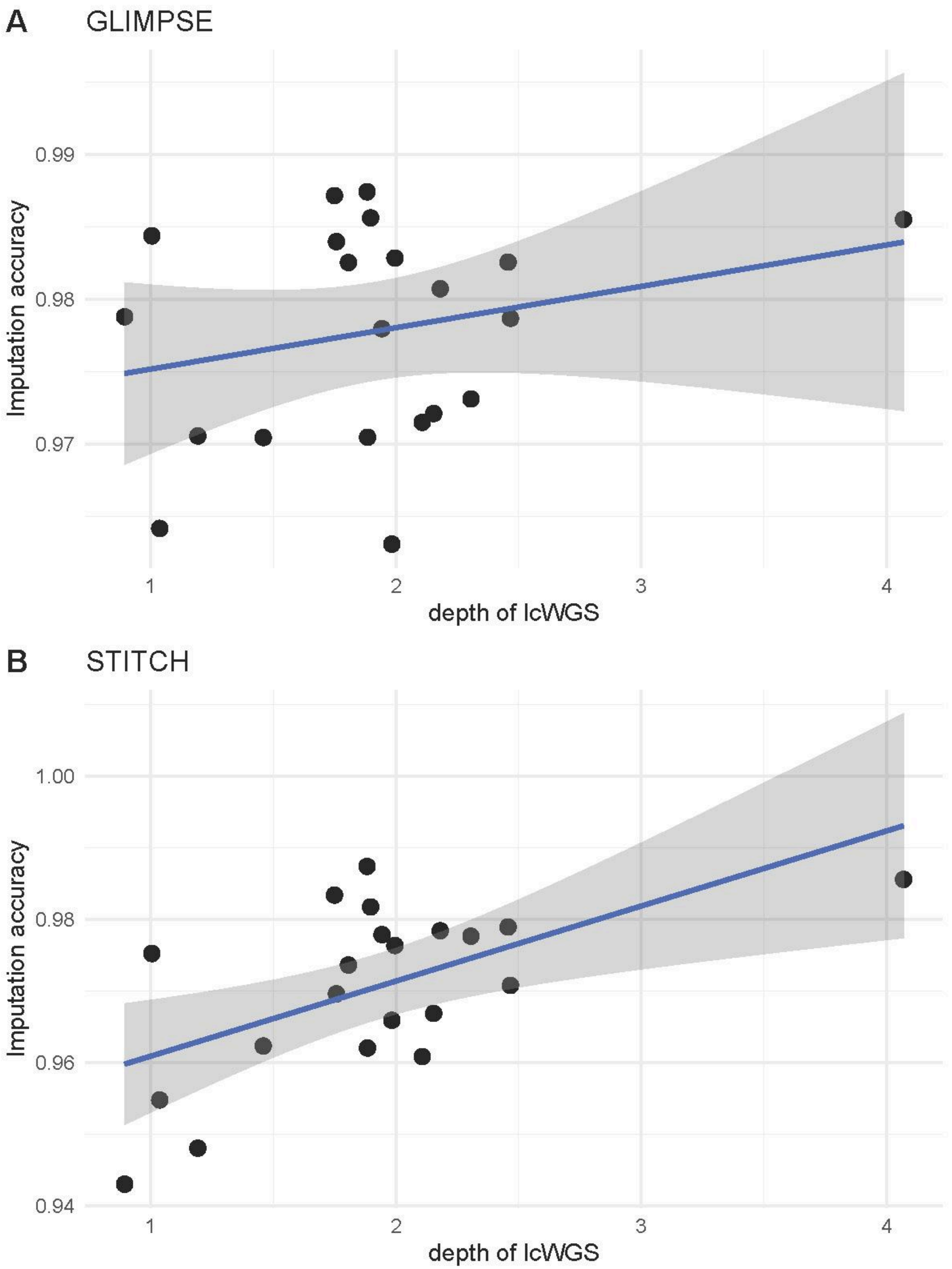

Supplementary Figure 11 - Imputation accuracy as a function of the lcWGS sequencing depth.

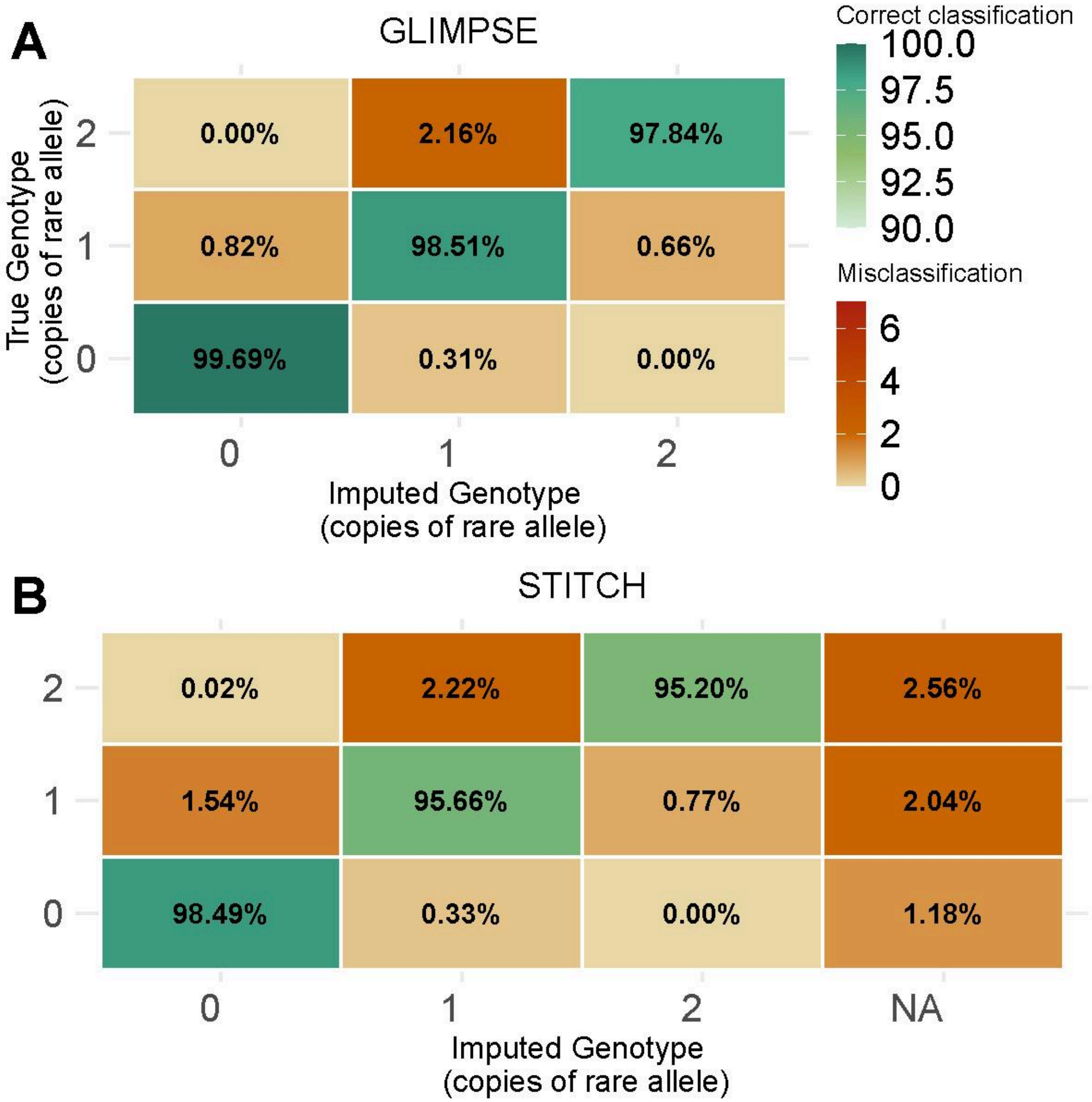

Supplementary Figure 12. Misclassification profile across all allele frequencies for the two full datasets for INFO > 0.8.

For GLIMSPE Each row represents the true genotype for these markers and the column the imputed one. A)For GLIMSPE B) Confusion matrix for genotype classification for STITCH. In B the NA column represents uncalled genotypes in STITCH. Diagonals are colored differently since higher is better, than off-diagonal where lower is better. All statistics calculated along the whole autosomal genome

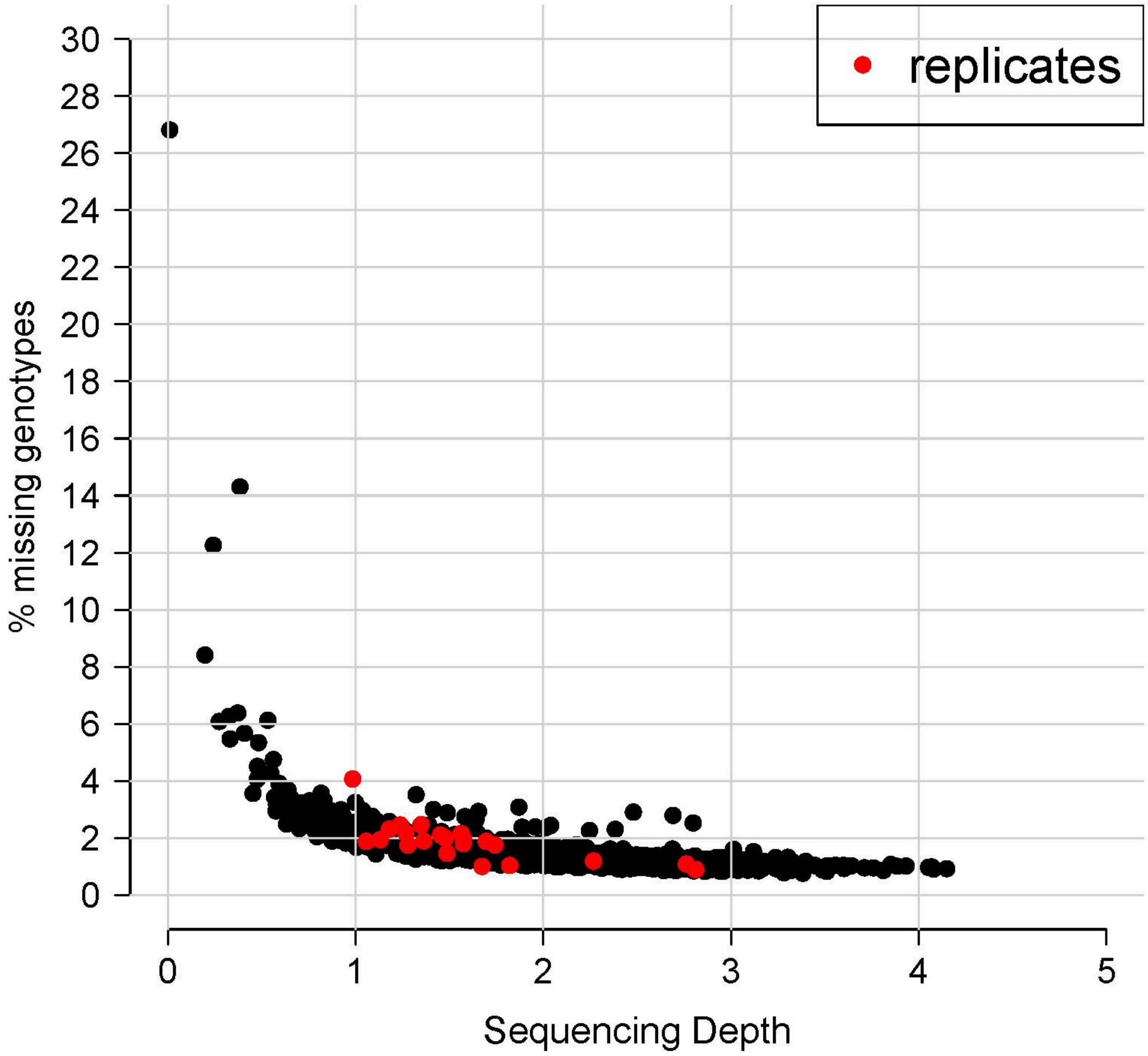

Supplementary Figure 13 - Missing rate per sample in STITCH as a function of the sequencing depth.
The n=21 replicates are highlighted with red.

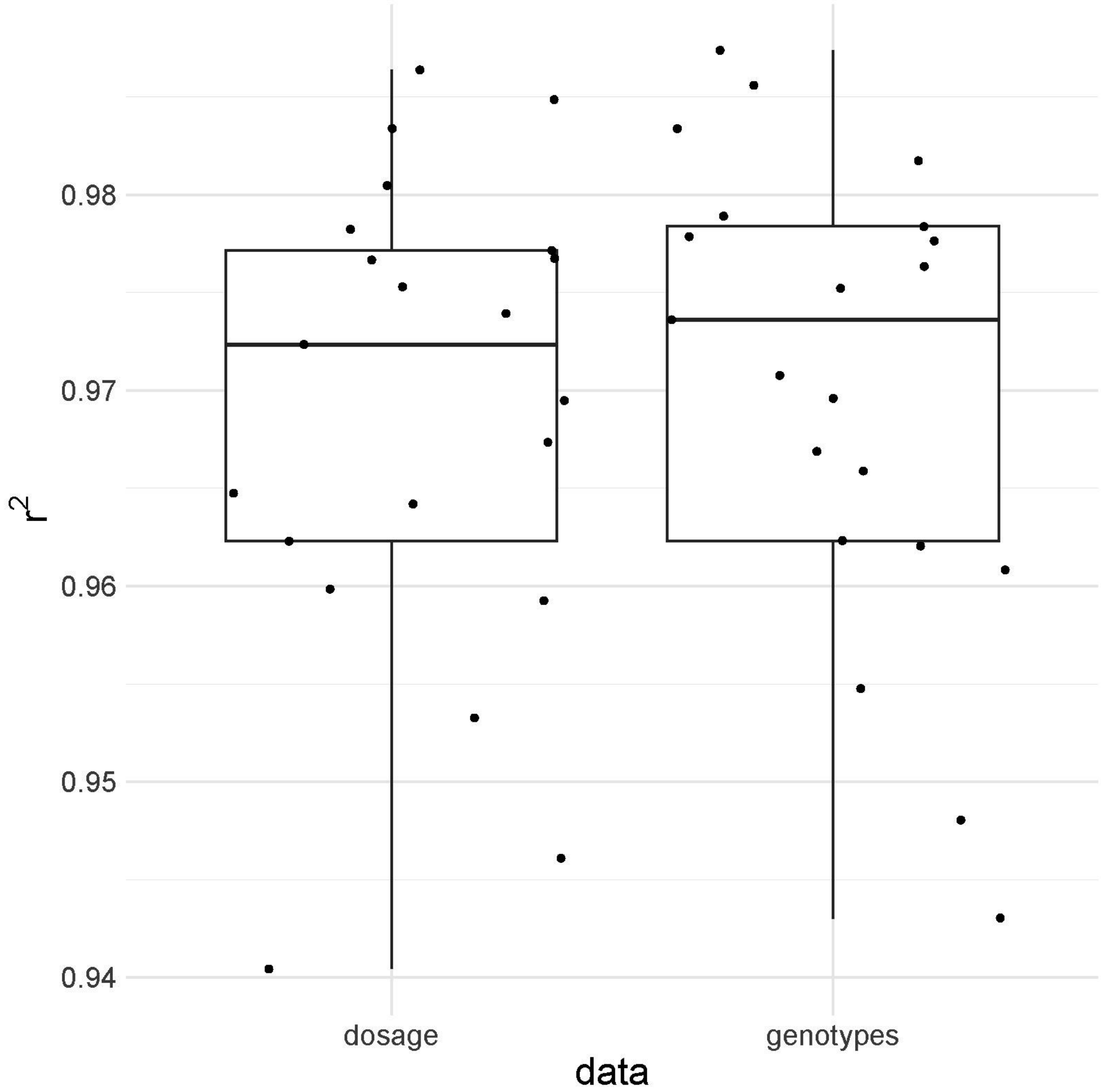

Supplementary Figure 14 - Imputation accuracy with STITCH when using or not the missing genotypes.
In ‘dosage’, we use the genotype dosage reported by STITCH which includes the genotypes set to missing by the software. On the contrary ‘genotypes’ use only the genotypes so it masks the missing data.

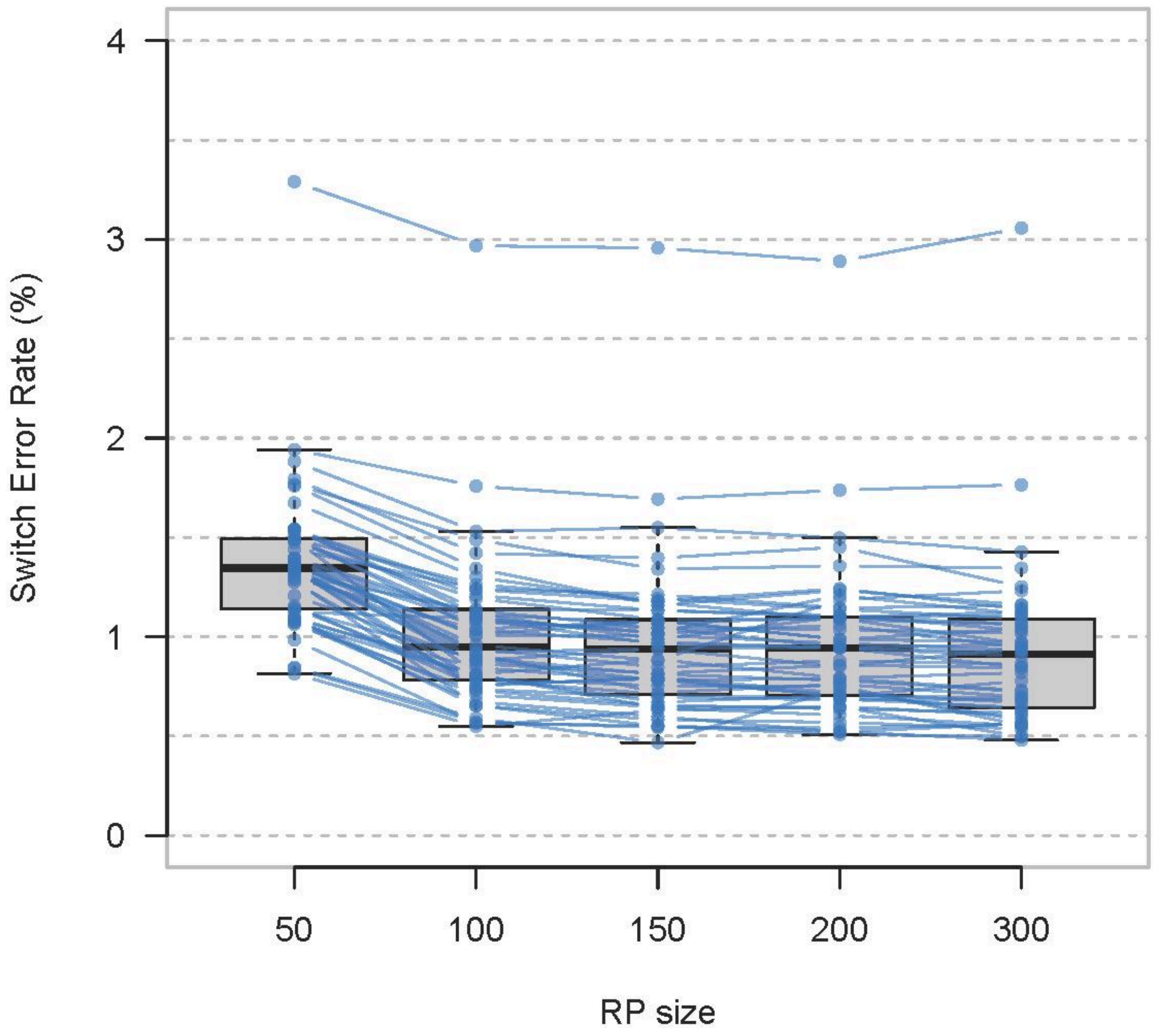

Supplementary Figure 15. Phasing errors in the subsampled reference panels.
Each line is one of the 50 individuals that were present in all reference panel sizes.

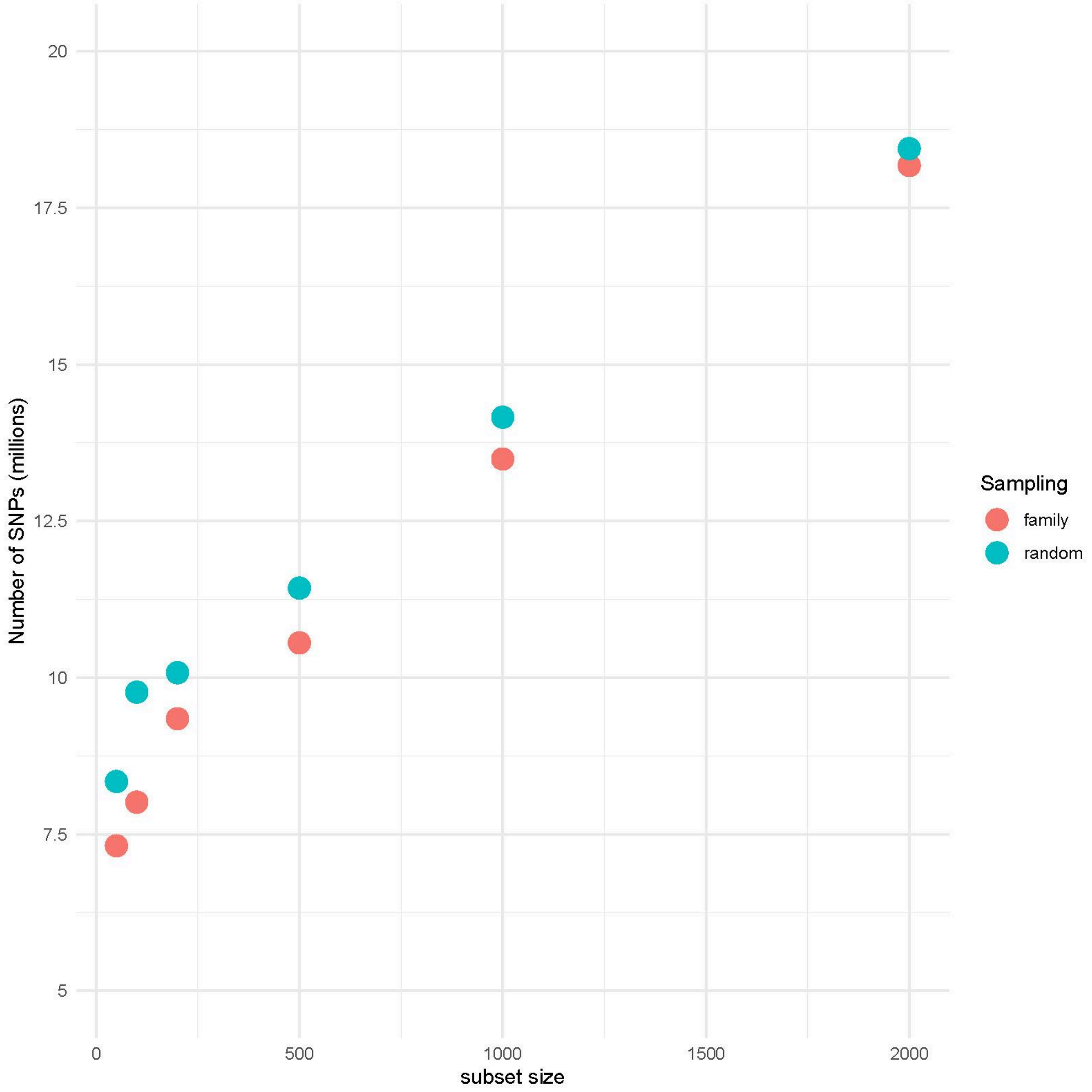

Supplementary Figure 16 - Number of bi-allelic SNPs discovered in each subset.

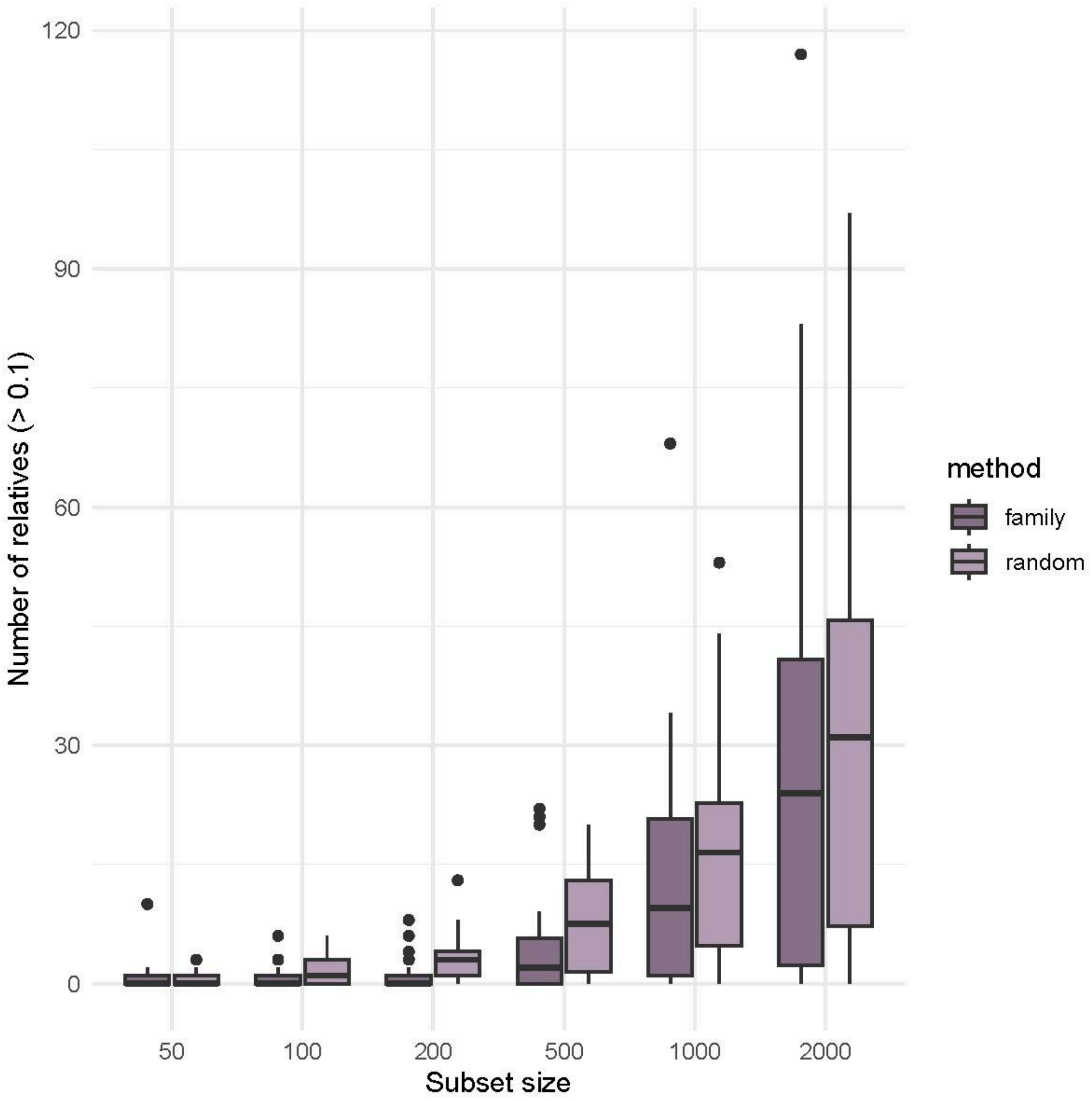

Supplementary Figure 17. The Number of ‘close’ relatives (relatedness >0.1) in each lcWGS subset dataset. l

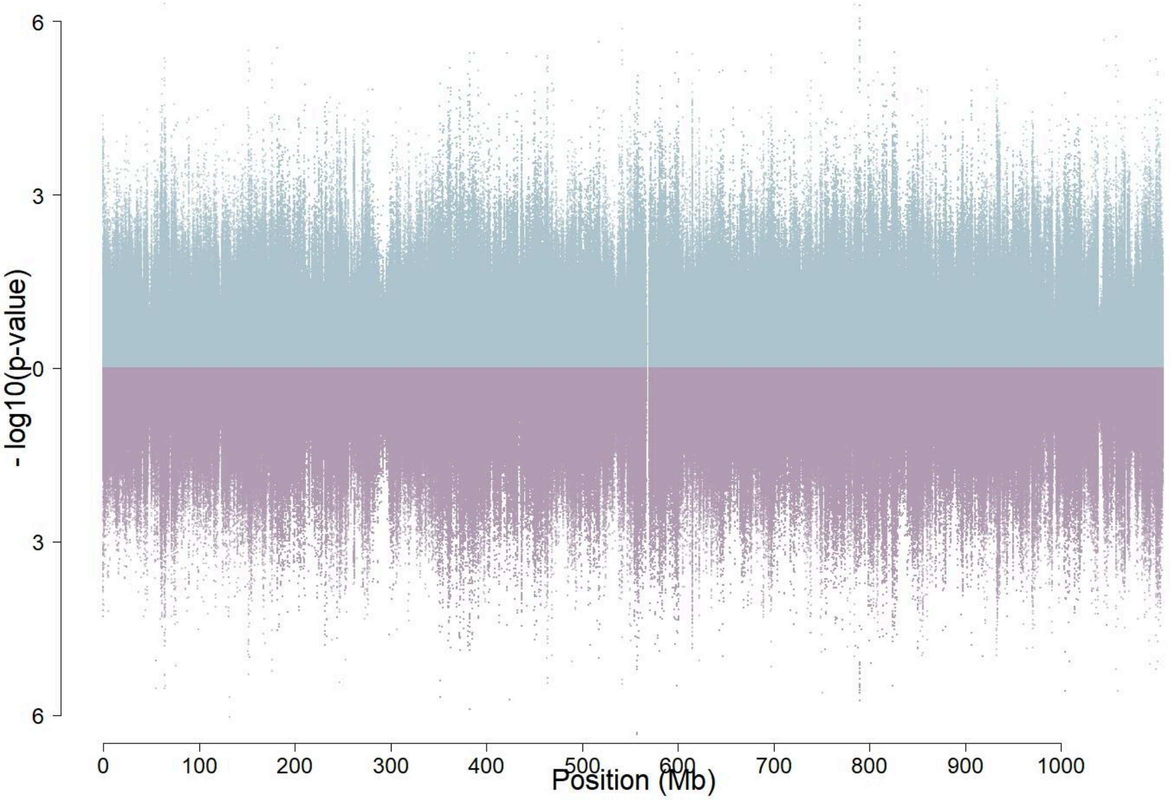

Supplementary Figure 18.
The whole genome GWAS on the size of the left tarsus of adult birds N=1980. Dots above the x-axis represent estimates with GLIMPSE. Dots below the x-axis represent estimates with STITCH.

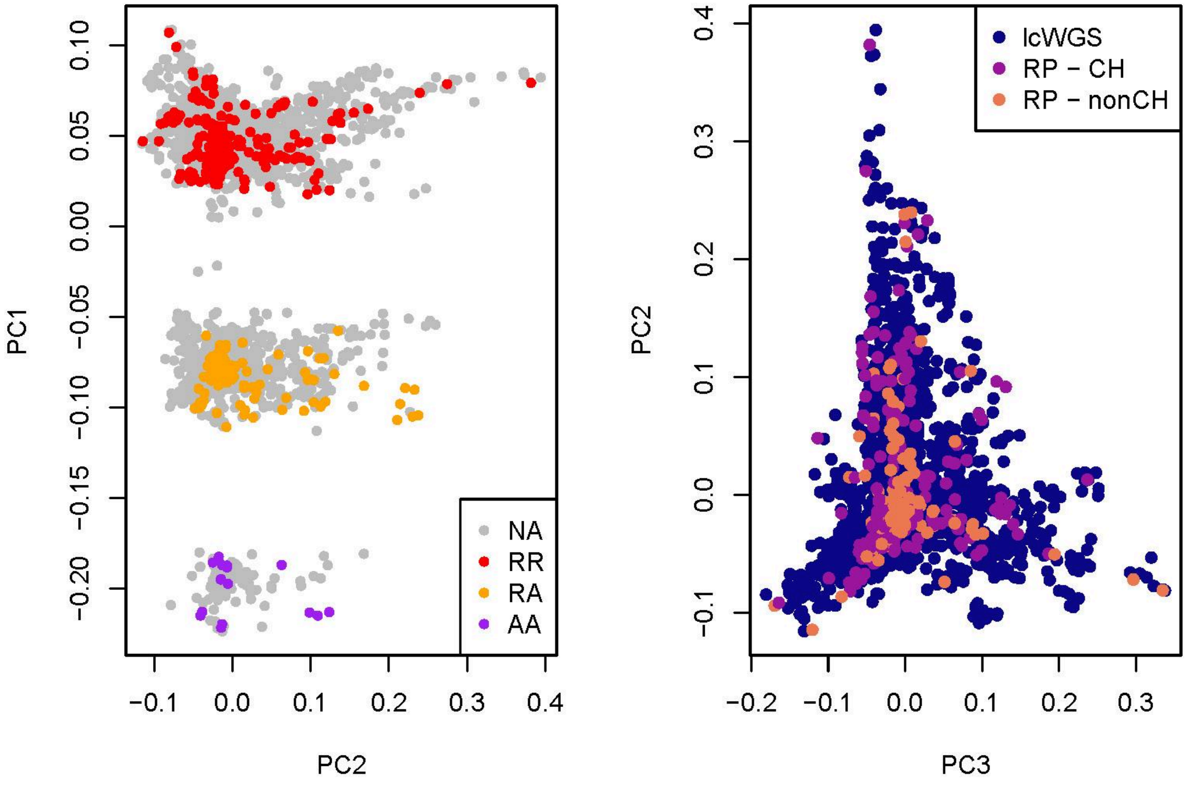

Supplementary Figure 19. Principal coordinate analyses of the samples used. This PCoA based on 1M random SNPs in the GLIMPSE-imputed dataset of 2800 lcWGS and the reference panel of 502 samples, shows the genetic variation within and between datasets. **Left**: PCo1 describes a known inversion in the system (Corval et al., 2023) with known genotypes highlighted as RR - homozygous reference, RA - heterozygotes , AA - homozygotes for the alternative allele. **Right**: lcWGS is the dataset of 2800 owls from Switzerland sequenced at 2x, RP - CH are the 346 reference panel samples from Switzerland , while RP - nonCH the 156 ones from the rest of the populations.

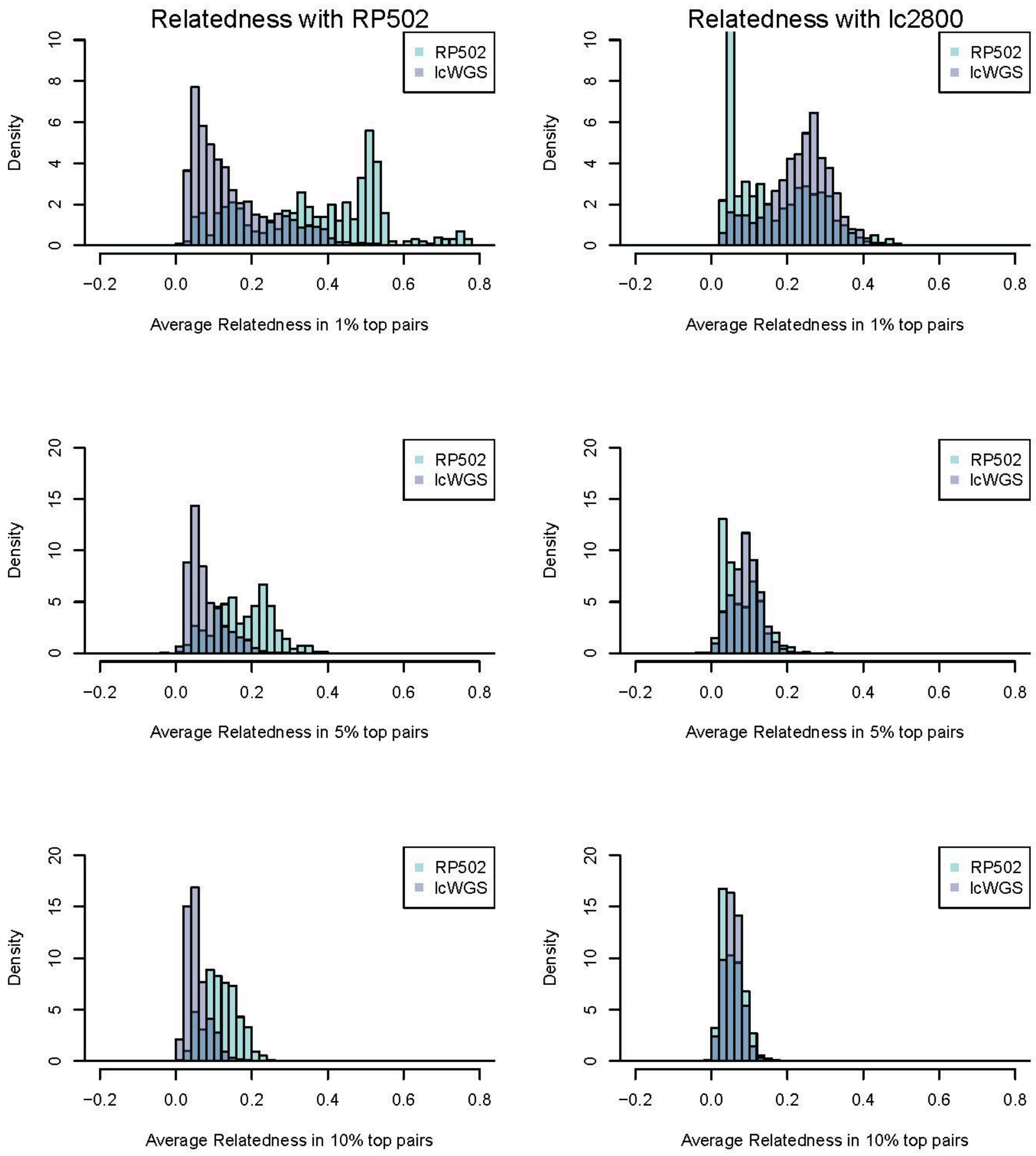

Supplementary Figure 20. A summary of the relatedness in our dataset.
Left) The average relatedness of each sample with their 1%, 5% and 10% closest relatives in the reference panel. The proportions should correspond roughly to 5, 25 and 50 individuals. **Right)** Similarly but for the lcWGS dataset of 2800 individuals. For each column we include a histogram for each dataset.

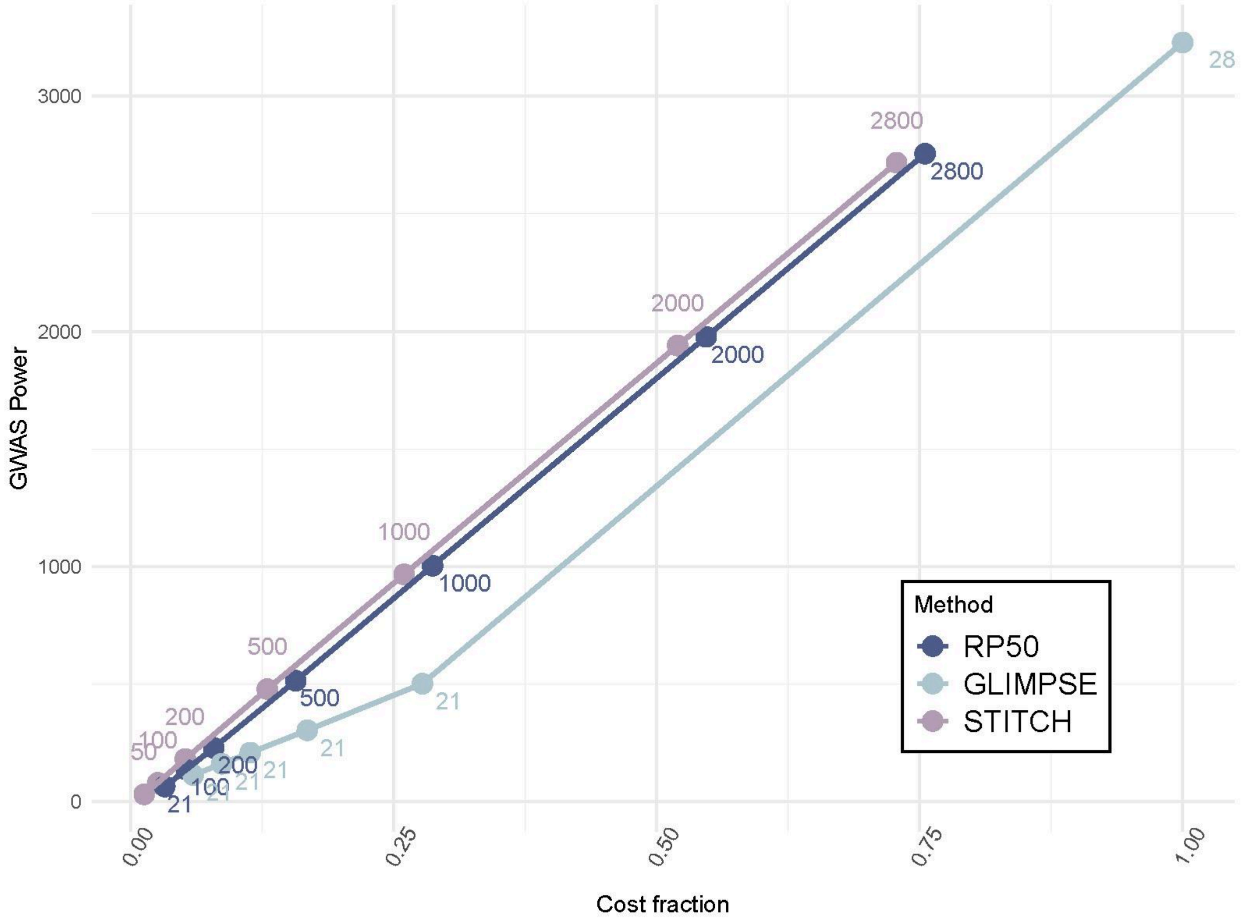

Supplementary Figure 21. The Power in a GWAS study as a function of data generation costs

GWAS power defined as effective sample size in a GWAS study (number of samples times (x) imputation accuracy). Cost estimated as: lc x (2 x gc + lc_{lib}) + hc x (15 x gc + hc_{lib}) and divided by the total. Lc is the size of the low-coverage dataset, gc the price of an owl genome at 1x sequencing coverage using an Illumina NovaSeq X machine in early 2026 (local estimate), lc_{lib} the price of a library prepared for lcWGS using SeqWell by Plexwell, hc the sample size of the reference panel, hc_{lib} the price of a library prepared for high coverage sequencing using TruSeq. RP50 refers to a reference panel of 50 samples, the lc size is printed by the points.

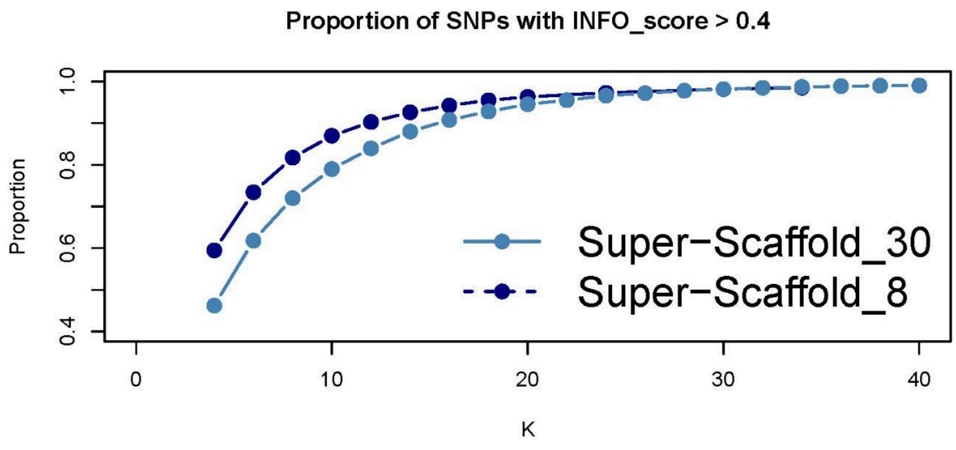

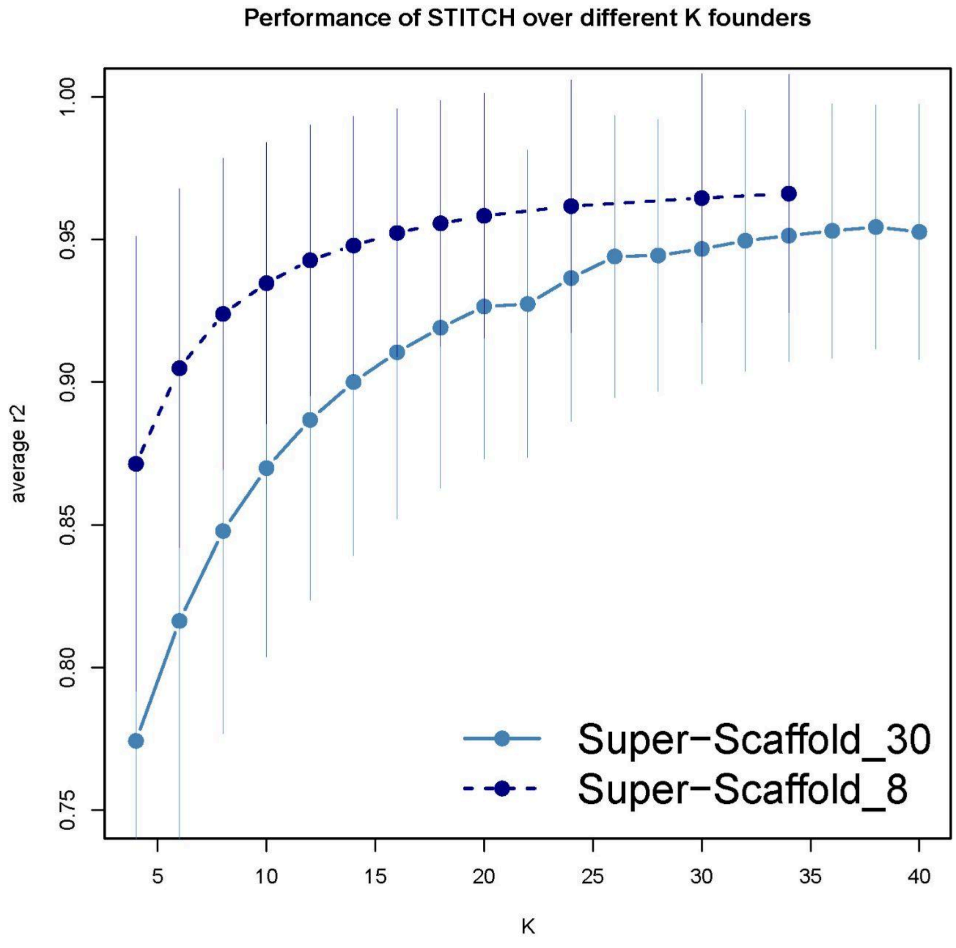

Supplementary Figure 22. Choosing the right K in STITCH.

Top panel shows the proportion of SNPs, relative to the total number of SNPs, with an information score larger than 0.4. The bottom panel shows the imputation accuracy of the 32 replicates for different values of K for each scaffold.

Supplementary Figure 19 - Relationship between imputation accuracy and scaled imputation accuracy.

Scaled imputation accuracy scales the dosage vector by the mean dosage and divides by the standard deviation.

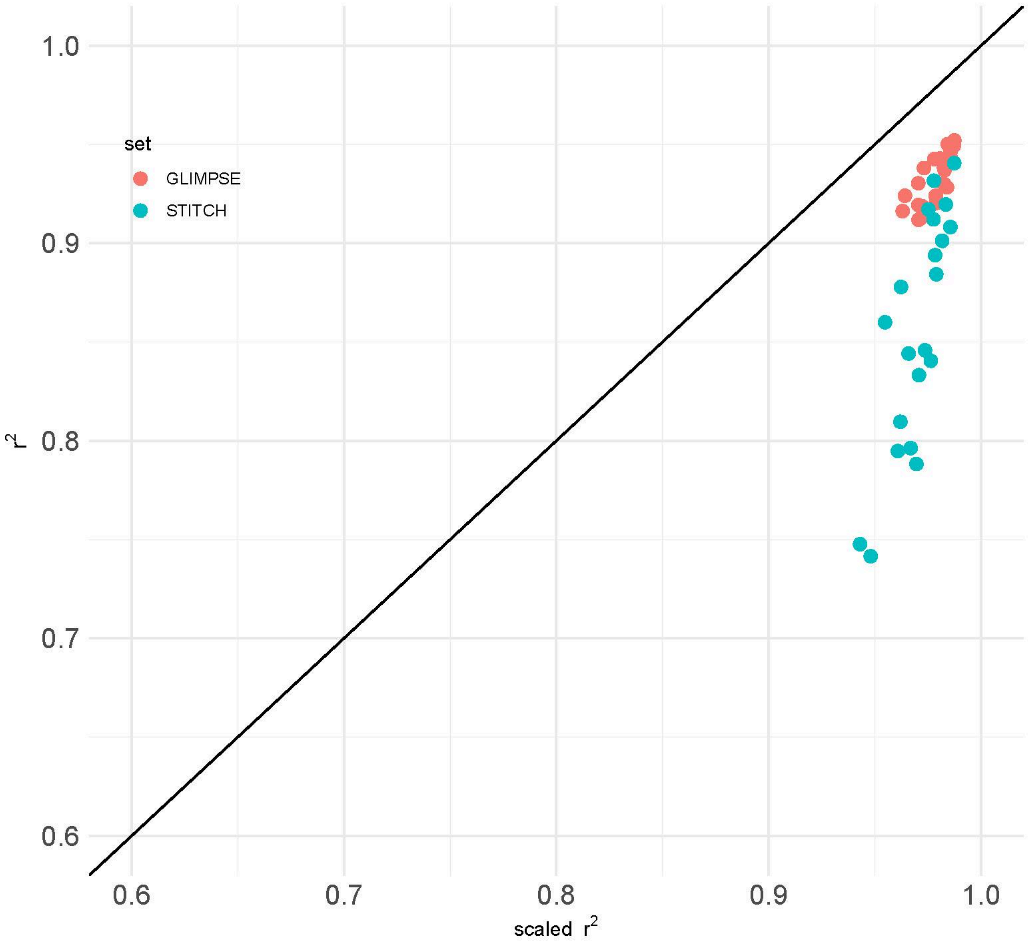

Supplementary Figure 23. Comparison of r2 and scaled r2.
Scaled r2 gives more weight to rare alleles.

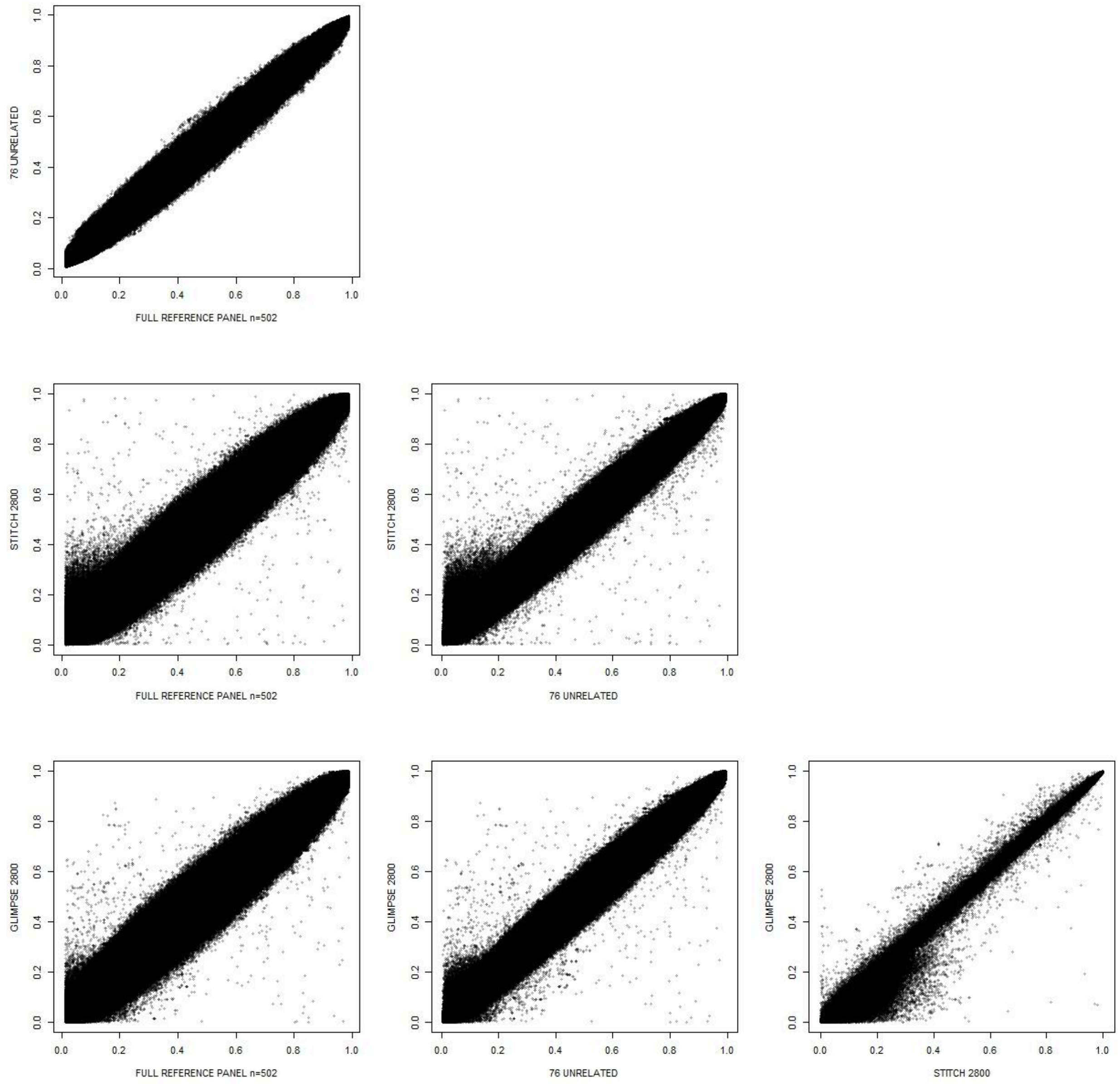

Supplementary Figure 24. The comparison between allele frequencies estimated with different datasets

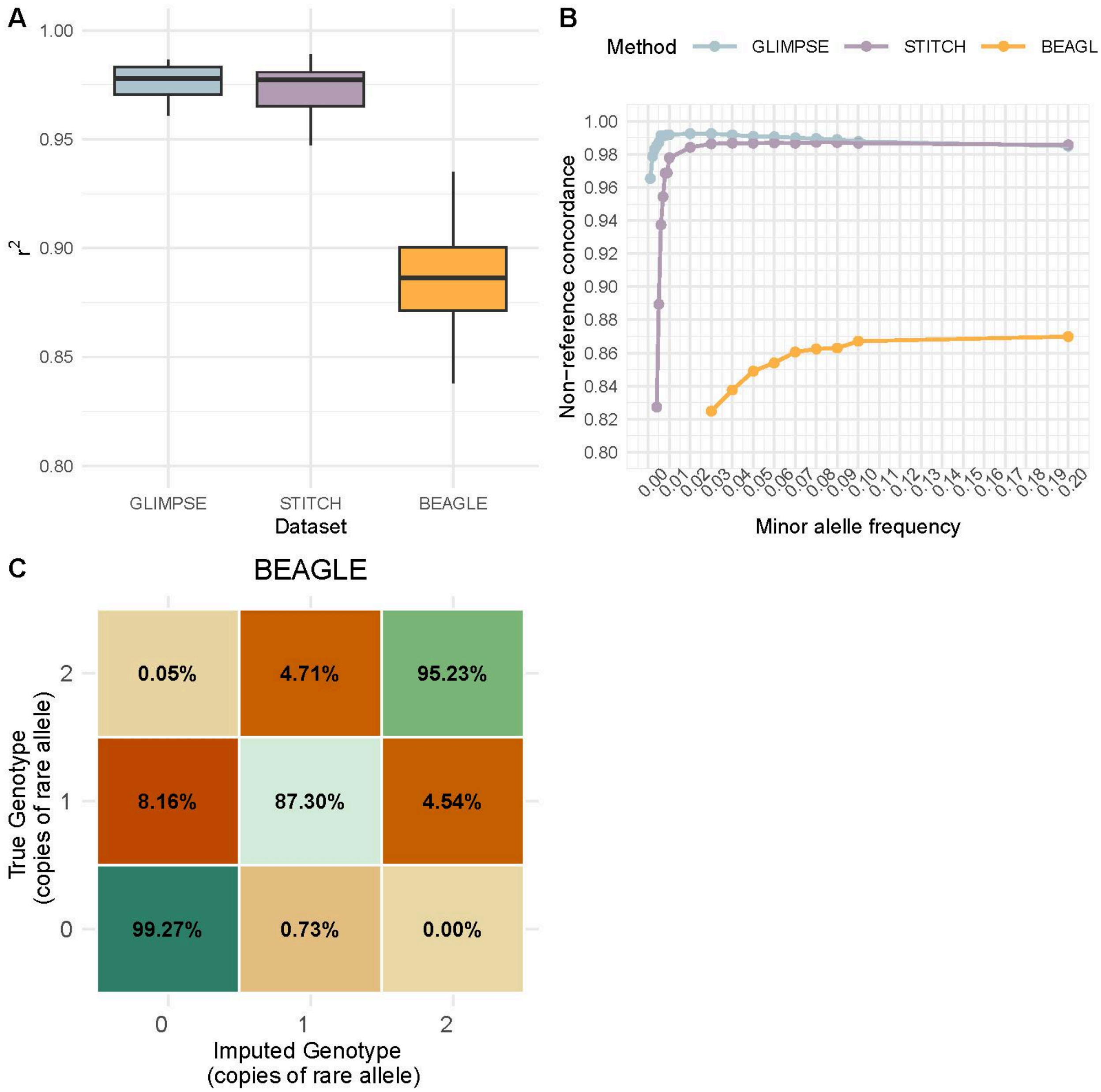

Supplementary Figure 25. The imputation accuracy including the BEAGLE dataset. A) Per sample imputation accuracy. B) The non-reference concordance (NRC) along different low-frequency alleles (effects estimated per SNP instead of in bins as in main text). C) The overall misclassification matrix when using BEAGLE.
